# Consideration of pathways for immunotoxicity of per- and polyfluoroalkyl substances (PFAS)

**DOI:** 10.1186/s12940-022-00958-5

**Published:** 2023-02-22

**Authors:** Veronika Ehrlich, Wieneke Bil, Rob Vandebriel, Berit Granum, Mirjam Luijten, Birgitte Lindeman, Philippe Grandjean, Andreas-Marius Kaiser, Ingrid Hauzenberger, Christina Hartmann, Claudia Gundacker, Maria Uhl

**Affiliations:** 1 Environment Agency Austria (Umweltbundesamt GmbH), Spittelauer Lände 5, 1090 Vienna, Austria; 2 Centre for Safety of Substances and Products, National Institute for Public Health and the Environment (RIVM), Bilthoven, The Netherlands; 3 Centre for Health Protection, National Institute for Public Health and the Environment (RIVM), Bilthoven, The Netherlands; 4 Division of Climate and Environment Health, Norwegian Institute of Public Health, Oslo, Norway; 5 Department of Public Health, University of Southern Denmark, Odense, Denmark; 6 Department of Biomedical and Pharmaceutical Sciences, University of Rhode Island, Kingston, RI USA; 7 Center for Pathobiochemistry and Genetics, Medical University of Vienna, Vienna, Austria

**Keywords:** Per- and polyfluoroalkyl substances, Immune function, Immunotoxicity, Molecular mechanisms, Vaccination response, HBM4EU

## Abstract

**Background:**

Per- and polyfluoroalkyl substances (PFAS) are of public health concern, because of their ubiquitous and extremely persistent occurrence, and depending on their structure, their bio-accumulative, mobile and toxic properties. Human health effects associated with exposure to PFAS include adverse effects on the immune system. In 2020, EFSA (the European Food Safety Authority) defined adverse effects on the immune system as the most critical effect for human health risk assessment, based on reduced antibody responses to childhood vaccines and similar effects observed in experimental animal studies. Likewise, the U.S. EPA (Environmental Protection Agency) considers PFAS-induced immunotoxicity, especially in children, as the critical effect for risk assessment. However, the mechanisms by which antibody concentrations are impacted are not completely understood. Furthermore, other targets of the immune system functions have been reported in the literature.

**Objective:**

The aim of this review is to explore PFAS-associated immune-related effects. This includes, relevant mechanisms that may underlie the observed effects on the immune system, immunosuppression as well as immunoenhancement, such as i) modulation of cell signalling and nuclear receptors, such as NF-κB and PPARs; ii) alteration of calcium signalling and homoeostasis in immune cells; iii) modulation of immune cell populations; iv) oxidative stress and v) impact on fatty acid metabolism & secondary effects on the immune system.

**Methods:**

A literature research was conducted using three databases (Web of Science, PubMed, and Scopus), which were searched in July 2021 for relevant studies published in the time frame from 2018 to 2021. In total, 487 publications were identified as potentially eligible and following expert-based judgement, articles relevant for mechanisms of PFAS induced immunotoxicity are discussed.

**Conclusions:**

Taken together, we show that there is substantial evidence from both in vitro and in vivo experimental as well as epidemiological studies, supporting that various PFAS, not only PFOA and PFOS, affect multiple aspects of the immune system. Timing of exposure is critical, because the developing immune system is especially vulnerable to toxic insults, resulting in a higher risk of particularly adverse immune effects but also other organs later in life.

## Introduction

Exposure to environmental toxicants, such as per- and polyfluoroalkyl substances (PFAS), can lead to substantial adverse effects on the immune system.

PFAS are a diverse group of chemicals, recently defined by the Organisation for Economic Co-operation and Development (OECD) as any fluorinated substance that contains at least one fully fluorinated methyl or methylene carbon atom without any hydrogen, chlorine, bromine, or iodine atom attached to it [1] consisting of thousands of individual compounds. Due to their amphipathic (hydrophilic and hydrophobic) properties, PFAS have been used in a wide range of applications and products for many decades [2, 3]. All PFAS are either intrinsically extremely persistent by or are transformed into extremely persistent ones in the environment [4] or within mammals [5, 6]. In addition, several PFAS have been proven to be bio-accumulative and toxic [7]. Although PFAS have been used commercially since the 1950s, particular concern about potential adverse human health effects grew in the early 2000s with the detection of considerable levels of perfluorooctanoic acid (PFOA) and perfluorooctanesulfonic acid (PFOS) in human blood and wildlife. Both substances are the most studied PFAS so far.

In 2016, the National Toxicology Program (NTP) of the US Department of Health and Human Services concluded that PFOA and PFOS are presumed to be immune hazards in humans based on strong evidence showing suppression of antibody responses from vaccinations in experimental animals and moderate evidence for suppression of antibody responses in humans [8]. Whilst PFOA and PFOS may both suppress the antibody response thereby exerting overlapping types of immunotoxicity, there are some differences. In addition to the suppression of antibody response, PFOS potentially suppresses natural killer (NK) cell function and might reduce disease resistance whereas, PFOA might also increase hypersensitivity [8]. In 2021, the US Agency for Toxic Substances and Disease Registry (ATSDR) reported that not only PFOA and PFOS, but also perfluorohexane sulfonic acid (PFHxS) and perfluorodecanoic acid (PFDA) serum concentrations are associated with a decreased antibody response to vaccines, as suggested by epidemiological evidence. Furthermore, there is limited evidence for perfluorononanoic acid (PFNA), perfluoroundecanoic acid (PFUnDA), and perfluorododecanoic acid (PFDoDA) for similar associations [9]. The European Food Safety Authority (EFSA) performed their risk assessment on the same health effects (reduced antibody response to vaccination in one-year-old children) on the sum of PFOA, PFNA, PFHxS and PFOS. A tolerable weekly intake (TWI) of 4.4 ng/kg body weight per week was derived [10]. Further, human studies published after EFSA’s scientific opinion reported an increased risk of infectious diseases, such as lower respiratory tract infections, thereby lending further support for the immunosuppressive effects of PFAS [11, 12]. However, a full understanding of the molecular mechanisms leading to PFAS-induced immunotoxicity has not yet been established due to various reasons. Particularly, the use of many different methods and models to investigate various types of immune responses for single members of the PFAS family have provided only mechanistic insights but not the complete picture. That being said, a lack of known mechanism(s) of immunotoxicity is not a requisite for setting exposure limits (e.g. for drinking water). Like EFSA, the U.S. Environmental Protection Agency (EPA) currently considers to use PFAS-induced immunotoxicity, especially in children, as the critical effect for risk assessment. The EPA defined interim updated health advisory values for drinking water for PFOS, PFOA, GenX chemicals (hexafluoropropylene oxide (HFPO) dimer acid and its ammonium salt) and PFBS (perfluorobutane sulfonate) which are even lower than EFSA’s TWI [13].

Major difficulties in evaluating health effects related to exposure to environmental toxicants often include insufficient mechanistic understanding and thus limits causal inference. Furthermore, current data requirements requested as part of a chemical legislative framework such as the REACH regulation do not align with the demands for assessing all key aspects of the immune system and its development [14]. Thus, due to a lack of evidence, many substances causing (developmental) immune effects may currently remain unnoticed in human hazard and health risk assessments, even though the developing immune system is a highly sensitive target for toxicity of environmental chemicals [14]. Critical windows of immune system development represent age-specific periods of prenatal and early postnatal development where irreversible maturational events of the immune system occur, such as seeding of peripheral tissues with lymphocytes or clonal selection of thymocytes in the thymus. Disruption or perturbation at these critical junctures can potentially result in both immediate and long-term adverse health effects in the developing child as well as the adult [15, 16]. Dynamic changes in the perinatal period before and just after birth include the basic maturation and distribution of immune cell types, and selection against autoreactive lymphocytes. In the perinatal period, the immune balance must change from protecting the foetus from immune-mediated miscarriage towards the ability to combat childhood diseases [17]. Substances like PFOA and PFOS can cross the placental barrier [18, 19], and have been detected in umbilical cord blood, breast milk and plasma samples of breastfed toddlers, indicating that maternal transfer occurs pre- and postnatally [9, 10]. It was estimated, that the median daily intake of the sum of PFOA, PFNA, PFHxS and PFOS for European infants and toddlers via food ranged from 0.84 to 12.2 ng/kg body weight per day at the lower bound (LB) and from 38.5 to 115 ng/kg body weight per day at the upper bound (UB), whereas the values for older age groups (adolescents, adults, elderly, very elderly) ranged from 0.42 to 3.1 ng/kg body weight per day at LB and from 11.4 to 41.5 ng/kg bw/day at UB. Given the early-life exposures, the effects of PFAS on the developing immune system are highly relevant for human health risk assessment [10, 20].

A recent example of the recognition of the vulnerability of the developing immune system as a target for toxicity is the draft opinion on health risks related to the presence of bisphenol A (BPA) in foodstuffs, proposing to lower the tolerable daily intake (TDI) for BPA by a factor of 100,000 [21]. The proposed TDI is based on an increase in T-helper 17 (Th17) cells, which are pivotal in cellular immune mechanisms and, among others, involved in the development of allergic lung inflammation and other inflammatory tissue responses.

Due to the various concerns related to PFAS, a number of policy measures have been taken for certain PFAS, such as inclusion in the International Stockholm Convention on POPs [22], regulatory measures under the REACH legislation of the European Union including the proposal for a wide-range restriction for all PFAS [23, 24]. Also, the overarching objectives of the Chemicals Strategy for Sustainability and the zero-pollution ambition under the European Green Deal address the restriction of use of PFAS [25].

Within the European Human Biomonitoring Initiative HBM4EU (www.hbm4eu.eu), human biomonitoring (HBM) studies in Europe have been collected and coordinated to address policy questions related to exposure, health effects and risks of various groups of chemicals, including PFAS. In this context, we conducted this review, with the aim to explore and describe PFAS-associated effects on immune function and the potential mechanisms involved.

## Methods

**Fig. 1:**
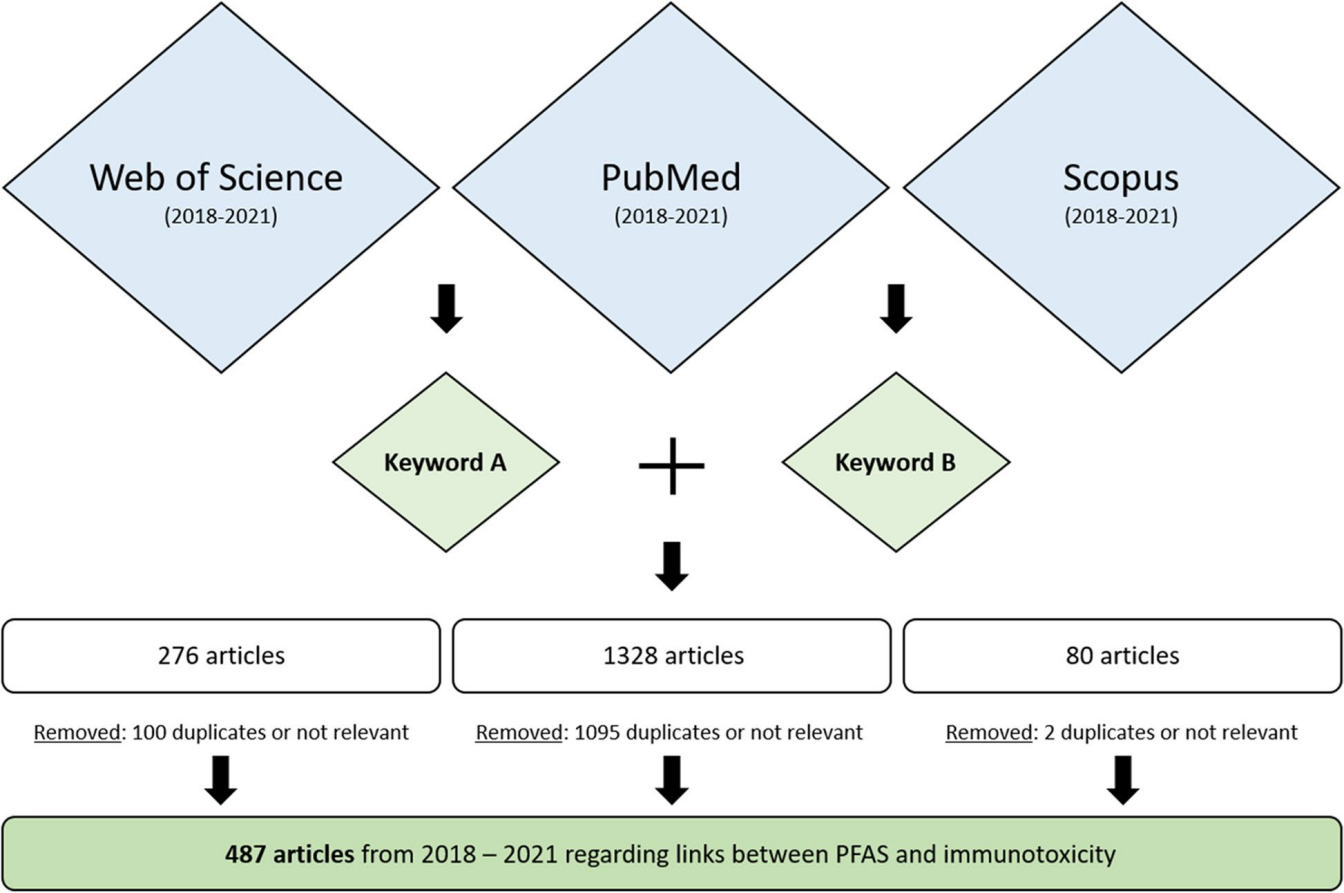
Illustration of the literature research. Figure Legend: For details on the search terms, please see Table 1 in Appendix

A systematic literature research was conducted using three databases (Web of Science, PubMed, and Scopus), which were manually searched in June and July 2021 for relevant studies published in the time frame from 2018 to 2021. Two keyword combinations (see Fig. 1) were used repeatedly, including the chemical name (i.e., perfluor*, polyfluor*, PFAS, PFBS, PFHxS, PFOS, PFBA, PFHxA, PFOA, PFNA, PFDA, PFUnDA, PFDoDA, HFPO-DA and GenX – corresponding to keyword A in Fig. 1) and the effect (e.g., immune*, asthma, apoptosis, NF-κB, PPAR, TDAR, … - corresponding to keyword B in Fig. 1). For the publications identified, titles and abstracts were manually screened, and publications considered out of scope (i.e., articles that did not contain any toxicological or epidemiological information concerning PFAS and immunotoxicity) were excluded. In total, 487 publications were identified as potentially eligible and tabulated. More details on the literature search are provided in Table 1 in Appendix. The information retrieved was subsequently reviewed by the authors and has been used for the present review based on expert judgement. Selection of the scientific papers for inclusion or exclusion was based on consideration of the extent to which the study was relevant to the mechanism of immunotoxicity of PFAS and general study quality considerations. Studies published in abstract form only (grant awards and conference abstracts) were not included. In addition, key studies already published before 2018 are discussed.

When deemed relevant, studies before 2018 were collected from existing literature reviews (NTP, ATSDR and EFSA) [8–10] for possible inclusion in the respective sections, to provide a complete picture on the potential mechanistic aspects of PFAS-induced immunotoxicity.

### Epidemiological studies

Separate literature searches on effects on the immune system in humans were performed in August 2021 (Medline) and January 2022 (PubMed) to retrieve papers not included in the EFSA opinions on PFAS [10, 26], as well as papers published after July 2019 for the 27 PFASs included in the EFSA 2020 Opinion [10]. Table 2 in Appendix lists details for search criteria for epidemiological studies. After the first screening of titles and abstracts, a total of 19 publications were identified as potentially eligible (inclusion criteria: human studies reporting on immune outcomes; exclusion criteria: not original paper, poster or congress abstract, and case studies). These publications were evaluated based on study design (ranking: randomised controlled trials (RCT), longitudinal observational studies, case-control studies, cross-sectional studies), blinding procedures in RCTs, the reporting of outcomes, population size and choice of study population, and statistics (power analysis, statistical methods, confounders). Finally, an evaluation was performed on whether the new studies strengthened or weakened the conclusions made by EFSA in 2020 [10] and if data on additional PFAS followed the same patterns.

## Results

### Knowledge on immunomodulatory effects of PFAS from epidemiological studies

This section aims to give an overview on the known effects in humans, sections 3-7 (Effect on key cell populations relevant for antibody production and cytokine modulation ff) provide a deeper mechanistic insight into immunotoxicity caused by PFAS.

EFSA has published two opinions on PFAS exposure and human health, covering exposure to 25 different PFAS [10, 26]. Several studies on vaccination responses were reviewed [27–35]. Overall, different PFAS measured at different developmental stages showed statistically significant inverse relations to vaccination specific antibody responses across the studies. After EFSA’s report from 2020, three new studies on vaccination responses [36–38], and a systematic review on PFAS and vaccination response have been published [39]. Due to different study designs, there appear to be some differences between studies, but they all report inverse trends between PFAS levels and vaccine antibody levels, hence supporting EFSA’s conclusion. Taken together, the studies show the strongest associations in children, although findings in adults are also notable, especially in temporal relation to a vaccination booster [31]. Given that substantial changes in serum-PFAS concentrations during and after the breastfeeding period have been observed, the time of exposure seems to be important [10]. Also, van Beijsterveld and co-workers report median plasma levels in > 200 Dutch infants at age 3 months (during breastfeeding: 3.080 [1.97–4.44] ng/ml for PFOA and 1.829 [1.26–2.89] ng/ml for PFOS) and at age 2 years (after breastfeeding) 2.360 [1.57–3.28] PFOA and 1.667 [1.04–2.34] PFOS), and confirm that breastfeeding is an early exposure pathway of PFOA and PFOS [40].

Most studies suggest that prenatal PFAS exposure is strongly related to an immune deficit, but early postnatal exposure may also be of importance, as is the cumulative exposure to PFAS at the time of vaccination [28].

When it comes to the effects of PFAS exposure on common infectious diseases, reported findings on upper respiratory tract and gastrointestinal infections were inconsistent. However, the evidence for an increased risk of lower respiratory tract infections (LRTI) is stronger. Overall, three studies have reported a lack of association between PFAS exposure and LRTI [34, 41, 42], potentially due to imprecise exposure assessment, while five prospective studies have reported positive associations [12, 43–46]. Additionally, three further studies on COVID-19, where the most affected organ is the lung, observed a positive association between PFAS exposure and confirmed COVID-19 cases (incidence and severity) [47–49].

Studies on asthma show no or inconsistent associations with PFAS exposure in children or young adults. In prospective studies, only very few statistically significant findings have been reported, but the type of PFAS and the direction of the response varies between the studies [35, 42–45, 50–53]. However, six cross-sectional studies reported an increased risk of asthma [44, 50, 54–57], whereas two further studies did not observe any statistically significant associations [32, 58]. It can be hypothesised that the increased risk of asthma seen in the cross-sectional studies may reflect an exacerbation of pre-existing disease, while PFAS may play a minor role in disease development. One study showed an increased risk of PFAS-associated asthma in children who had not received the MMR vaccination, thus suggesting a possible cofactor [59]. For PFOS and PFOA, EFSA stated, that epidemiological studies provide insufficient evidence to conclude on associations between exposure to PFAS and asthma [10]. From investigations conducted in the context of HBM4EU, linking human biomonitoring and health effects, the association between PFAS exposure and asthma was also not considered consistent across studies [60]. In addition, due to too few studies and inconsistent findings, no conclusion can be drawn with regard to the effect of PFAS exposure on lung function, atopic dermatitis, rhinitis, and allergic sensitisation (measured as serum IgE levels or in skin prick tests).

### Repeated dose toxicity and immunotoxicity studies in animals

This section provides an overview of functional immunotoxicity tests in experimental animals, as well as studies that investigated the resting immune system; sections 3-7 provide a deeper mechanistic insight into immunotoxicity caused by PFAS. Twenty-three functional immunotoxicity studies and 14 studies that investigated the resting immune system are discussed in this section. Of these studies, eight are from our literature review (2018 and newer, see Methods) in addition to the discussion of the immunotoxicity studies already published before 2018.

Functional assays with laboratory animals provide direct evidence for immunosuppression upon exposure to PFAS, specifically by showing decreases in the T-cell dependent and independent antibody responses (TDAR and TIAR), and decreased disease resistance in host infection studies.

TDAR assays are used by regulatory agencies for the evaluation of the immunotoxic potential of pharmaceuticals and chemicals, as they are a robust and sensitive method and provide a functional readout of B-cells, T-helper cells as well as monocytes as antigen presenting cells [61]. A growing number of studies demonstrate that a positive response in the TDAR in exposed experimental animals is predictive of immunotoxicity in humans [62], with the analogous human response being antibodies generated towards a specific vaccine.

Table 3 in Appendix lists functional immunotoxicity studies (TDAR,TIAR, host resistance and lymphoproliferative response studies) performed with PFAS in rodents, with a focus on recent studies (since 2018). Dose-dependent decreases in serum IgM and/or IgG concentrations were seen in most studies for PFOA [63–69], PFOS [64, 70–75], hexafluoropropylene oxide-dimer acid (HFPO-DA) [76], and an aqueous film-forming foam (AFFF) mixture with serum concentrations of C5-C10 PFAS, including chlorinated polyfluorooctane sulfonate (Cl-PFOS) [77], but not in all studies [78–81]. For PFDA [82], PFHxS [80], and perfluoro-2-methoxyaacetic acid (PFMOAA), perfluoro-3-methoxypropanoic acid (PFMOPrA) and perfluoro(4-methoxybutanoic) acid (PFMOBA) [79], no changes in serum concentrations of immunoglobulins (IgMs, IgGs) were observed, although in Ramhøj [80] and Woodlief et al. [79] the positive controls (cyclophosphamide and PFOA, respectively) were also negative; therefore, these study outcomes were considered inconclusive.

Furthermore, the results obtained from a host infection study for PFOS [83] indicate immunosuppression, but another study was negative [81], as was the case for a study with PFDA [82]. Nevertheless, in cases where serum IgM or IgG levels were not impacted, other effects such as changes in specific cell populations in lymphoid organs [81] or alterations in lymphoid organ weights [82] were observed.

Experimental animal studies that investigated the resting immune system indicate that it is a relevant target for PFAS-induced toxicity. Immunological alterations include decreased lymphoid organ weights for PFBS [84, 85], PFOS [84], PFHxA (perfluorohexanoic acid) [86, 87], PFOA, PFNA, PFDA [86], PFUnDA [88], and PFDoDA [89], alterations in thymic and splenic lymphocyte subpopulations for PFOA [90], PFOS [90–92], and PFNA [93, 94], atrophy of the thymus and the spleen for PFBS [84], PFHxA [95], PFNA [86, 96, 97], and PFDA [82, 86], increased hypocellularity of the bone marrow for PFBS [84], PFOS [84], PFOA [86], PFNA [86], and PFDA [86] and atrophy of the mandibular and mesenteric lymph nodes for PFNA [86]. Although such parameters are only indicative and should not be used to conclude on immunotoxicity without considering the performance of functional tests, they add to the weight of evidence for PFAS-induced immunotoxicity [14].

In summary, the main body of TDAR studies with PFOA and PFOS demonstrates a robust pattern of findings to support PFOA- and PFOS-associated immunosuppression, although negative results also have been reported. The heterogeneity in the data can partly be explained by differences in the antibody response by species (mice vs. rats), outcome (primary vs. secondary response), and study protocol (dosing regime, exposure duration). Rats appear to be less susceptible (probably due to more rapid clearance) than mice to PFAS-associated antibody suppression. There is less data available on other PFAS and replacement products, but the outcomes of the TDAR studies available showed those PFAS were less potent or non-responsive, although in some studies the positive controls were negative. For some PFAS, such as PFBS, PFHxA, PFNA, PFUnDA, and PFDoDA, no TDAR studies have been performed at all, although triggers for immunotoxicity have been observed in repeated-dose experiments that warrant further functional testing according to the IPCS/WHO (International Programme on Chemical Safety by the World Health Organization) Guidance Document [14].

### Effect on key cell populations relevant for antibody production and cytokine modulation

#### Effects on cytokine release

The NTP (2016) described evidence that PFOS exposure was associated with a shift of cytokine balance away from Th1 cytokines (reduced secretion of IL-2 and INF-γ) and towards Th2 cytokines (increased secretion of IL-4) in mice exposed to higher doses (0.833 to 20 mg/kg/day). However, given the heterogeneity in study design, tissues, and cell populations investigated, it is difficult to evaluate whether or not there is a clear or consistent pattern for changes in these cell signalling molecules after exposure to PFOA or PFOS and the evidence was described as “inconclusive and variable” [8].

The effects of PFAS on cytokine homeostasis in humans and experimental animals remains poorly understood. In a TDAR study with mice exposed to PFOA an overall reduction of Th2 cytokines (significant: IL-5 and IL-13; non-significant: IL-4), a mixed response for Th1 cytokines (significant reduction of IL-12 and non-significant increase in IL-2 and IFN-γ) were observed by De Guise and Levin. This showed a favourable Th1 balance and a general decrease in pro-inflammatory cytokines (IL-17α, IL-1α: non-significant, IL-6 and a significantly increased TNF-α). The authors postulate a potential role for T helper (Th) cells in the immunotoxicity of PFOA [63]; study design is described in Table 3 in Appendix.

In a human study [98], 21 PFAS were identified in 198 Chinese women of childbearing age. In single PFAS models, PFAS were positively associated with Th1 and regulatory T-cell (Treg) cytokines, and negatively associated with Th2 and Th17 cytokines. The Bayesian Kernel Machine Regression (BKMR) model showed a significantly positive association of PFAS mixture with TGF-β and a negative association with IL-10. A deviation of the immune system from Th2 toward Th1 has been implicated in pregnancy complications, such as recurrent miscarriage, preeclampsia and foetal growth restriction. It should however be noted, that the effect of PFAS on cytokine homeostasis in humans is poorly understood [34, 55, 99–101]. Nian and co-workers point out, that evidence so far has been inconsistent, as also positive correlations between PFAS and Th2 cytokines in humans were shown [98].

In conclusion, in line with the conclusion made by Nian et al. [98], inconsistencies in the effects on Th1/Th2 cytokine levels exist between various studies. Unfortunately, there are no studies that identify the specific cell types involved or link the cytokine changes to the suppression of the antibody response. These cytokines have multiple physiological roles and may reflect inflammation rather than changes in antibody-related cell signalling.

#### Modulation of key cell populations – epidemiological studies

Immunophenotyping is a commonly used tool in immunotoxicity testing in animal studies and for evaluating drugs in clinical trials. One publication by Dong and co-workers [54] showing positive association between serum eosinophil counts among asthmatic cases across and PFAS concentrations in children is mentioned in previous reports of immunotoxic effects of PFAS by NTP and EFSA [8, 10]. An additional four epidemiological studies describe associations between PFAS exposure and peripheral white blood cell (WBC) counts [34, 102–104]. Two of these publications were identified in the literature update on epidemiological data [34, 104], and two earlier publications were added based on expert knowledge [102, 103]. The study by Oulhote et al. prospectively examined 56 children to determine associations between exposures to five persistent PFAS (PFOS, PFOA, PFHxS, PFNA, PFDA) since birth and the differential counts of WBCs [102]. Higher 18-month and 5-year PFAS concentrations were associated with increased basophil counts in the children at age 5 years.

Knudsen and co-workers [103] investigated the association between the sum of 15 PFAS and haematological markers in 189 Greenlandic pregnant women. The markers investigated included white blood cells, lymphocytes, neutrophils and monocytes, which were significantly inversely associated with the sum of PFAS suggesting an immunosuppressive potential of PFAS in pregnancy. However, as the blood samples of the women were taken in different trimesters and due to the physiological changes in immune cell concentrations during pregnancy, these findings need confirmation.

In the study by Abraham and co-workers (also described in section Knowledge on immunomodulatory effects of PFAS from epidemiological studies), associations between PFAS and several immune cell parameters in 1-year-old children following vaccination against Haemophilus influenza type b, tetanus and diphtheria were determined [34]. No changes in white blood cells counts or in main lymphocyte populations or CD4/CD8 cell ratios were associated with the PFAS measured. However, a positive association between PFOA and two phenotypic subpopulations (CD45RO+ CD45RA-; CD27-) among CD8+ T-cells was suggested. In lymphocytes stimulated ex vivo with tetanus or diphtheria toxoid a reduced IFNɣ production was associated with increased plasma PFOA levels. A similar PFOA associated reduction in IFNγ was not observed in response to the general immune stimulant PHA, suggesting an effect specific tothese vaccine antigens.

In the study by Lopez-Espinosa and co-workers, associations were described between PFAS exposure and peripheral WBC counts in a human population in the Mid-Ohio Valley, USA with drinking water exposure to PFOA and background exposure to other PFAS [104]. In this study, PFAS were positively associated with absolute lymphocyte count and the counts of T-cells, B-cells, and natural killer (NK) cells. However, no significant associations were reported for changes in the percentages of B, Th and Tc lymphocyte subsets. The strongest association with lymphocyte counts was seen for PFHxS and to a lesser degree PFOS followed by PFOA. No significant association with changes in CD4/CD8 ratios was found for these three PFAS.

#### Modulation of key cell populations - animal studies

This section discusses evidence available before 2018 (i.e. 11 studies), and two more recent studies (2018 and newer), were identified by our literature research (see Methods). In animals exposed to PFAS, changes in lymphocyte subpopulations have been reported, but the data shows variability in the changes observed between sexes, and across studies. In the NTP 28-day rat studies reported above [84, 86], haematological analysis suggested some dose-dependent changes in leukocyte counts. The most consistent observation was a reduction in eosinophils in both males and females. Reductions in total leukocytes and neutrophils in males were observed for PFOS and PFNA. In mice, a reduced number of thymocytes and/or splenic lymphocytes is reported in several studies following exposures to PFOS and PFOA [64, 65, 70–72, 93, 105, 106]. A decreased number of bone marrow B-lymphoid cells in response to PFOA exposure (0.002% w/w in diet) and PFOS (0.02% w/w in diet) has also been reported [107]. Based on data from a study by Dong and co-workers, a reduced TDAR response appears to be a more sensitive endpoint than reductions in splenic and thymic cellularity [72]. Furthermore, the reported direction of change, if any, in lymphocyte subsets varies between studies and sex.

In a developmental mouse study, splenic Treg numbers were reduced at the highest dose (2 mg/kg bw/day) and isolated CD4+ cells from adult offspring, exposed via the dams to PFOA during gestation and through weaning, secreted lower amounts of the immunosuppressive cytokine IL-10 than cells from controls in males only [108]. Gestational exposure (GD 1-17) to PFOS (5 mg/kg bw/day) led to a reduced number of thymic CD4+ cells in 8-week-old male offspring [74].

In conclusion, animal studies show that some PFAS, including PFOS and PFOA, can reduce splenic and thymic cellularity and levels of circulation WBCs. The few epidemiological studies that enumerate WBC subclasses examine different human populations and are insufficient to give a clear picture of potential effects on immune cell phenotypes of PFAS exposure levels relevant to humans.

### Modulation of nuclear receptors / cell signalling

Considering that gene expression is rarely dependent on a sole transcription factor, and that cross-talk between various transcription factors is known to widely occur, PFAS effects in rodents are probably a result of multiple interlinked pathways [109]. The EFSA panel [10] reviewed transactivation of several nuclear receptors, as observed from in vivo and in vitro studies, including PPARs (peroxisome proliferator-activated receptors), NF-κB (nuclear factor kappa B), CAR (constitutive activated receptor), Nrf2 (nuclear factor erythroid 2-related factor 2), PXR (pregnane X receptor) and RXR (retinoid X receptor). Although some of these nuclear receptors may have an indirect effect on immune health, the following sections were focussed on the modulation of NF-κB and PPARs. This selection was made because of the interactions of NF-κB and PPARs with the immune system, and data on other nuclear receptors are less conclusive.

#### Modulation of NF-κB regulated gene transactivation

NF-κB is found in almost all human and animal cell types and known to be involved in cellular responses to stimuli such as stress, cytokines, free radicals, heavy metals, ultraviolet irradiation, oxidized low density lipoprotein (LDL), and bacterial or viral antigens [110]. NF-κB signalling coordinates not only adaptive and innate immune responses but is also involved in the regulation of apoptosis. Depending on the context, NF-κB triggers either pro- or anti-apoptotic pathways and is thus involved in the decision of whether a cell may survive or die.

Once activated, NF-κB can induce the transcription of various genes and thereby regulate inflammation. NF-κB targets inflammation not only directly by increasing the production of inflammatory cytokines (e.g. IL-1, IL-2, IL-6, IL-8, IL-12, TNF-α), chemokines (e.g. MCP-1, IL-18, RANTES, MIP-2, CXCL1, CXCL10) and adhesion molecules (e.g. ICAM-1, VCAM-1, ECAM-1, MMPs), but also by regulating cell proliferation, apoptosis, morphogenesis, and differentiation [110].

A total of 27 experimental studies dealing with the modulation of NF-κB by PFAS. Of these, 21 studies showed activation of NF-κB, two studies inhibition of LPS-induced NF-κB activation, whereas one study showed opposite effects in high dose and low dose ranges, and three studies did not show any impact on NF-κB. Of the 27 studies listed, 11 studies originated from our literature research (see Methods) in addition to the discussion of the studies already published before 2018.

Table 4 in Appendix lists experimental studies reporting the modulation of NF-κB by PFAS. The majority of studies were performed with PFOA (*n* = 13) and PFOS (*n* = 12), and much less with other PFAS members, where PFDA and PFNA were tested in three studies, and PFBS, fluorotelomer (i.e., perfluorooctyl-ethanol- (8:2 telomer)), PFUnDA and PFHpA only in one study each. In contrast to the other PFAS studied, PFNA did not modulate NF-κB activity in any of the studies reported [93, 94, 111].

Lee and co-workers [111] suggest that the chain length determines the outcome, since PFDA (C10) and PFUnDA (C11) caused an increase in NF-κB activity, while the compounds PFNA (C9) and PFHpA (C7) had no effect on NF-κB activity. In zebrafish, higher doses of PFOA caused an increase in NF-κB activity while lower doses had an inhibitory effect [112].

In conclusion, the body of evidence indicates that in experimental studies most PFAS tested induce NF-κB, although contradictory effects have been observed. It may be possible to explain these discrepancies by the experimental model used, although there are indications that chain length and dosing schedule have an influence. Caution must be taken when interpreting rodent in vivo data in the case of PFAS, since next to higher exposure levels in rodents, exposure duration may also be divergent, i.e. several decades for humans versus several (2–14) weeks for animals [109]. Furthermore, in vitro data using (non-human) cell types not from immune lineages are not ideal for detecting effects as NF-κB and cytokine modulation.

#### Involvement of PPARs

Based on our literature review (see Methods) 13 studies were identified as relevant for this section. Peroxisome proliferator-activated receptors (PPARs) are a family of nuclear hormone receptors consisting of the three identified subtypes PPARα, PPARβ/δ, and PPARγ [113, 114]. They are expressed in various cells including those of the immune system and they have been described to interact with both the innate as well as the acquired immune system [115]. An excellent overview of the general role of PPARs concerning immune responses is provided by Christofides et al. [114].

The EFSA Panel stated in their assessment that modulation of PPARs may play a part in the immunotoxicity of PFAS. Nevertheless, a detailed understanding of the involvement of PPARs is lacking, and further research on this topic is necessary [10]. We evaluated experimental studies of recent years, which reported PPAR modulation by PFAS. Table 5 in Appendix lists the experimental binding/agonistic effects of PFAS to all three PPAR subtypes.

PPARα is a transcription factor that regulates lipid catabolism and inflammatory responses [116] by for example increasing the gene expression of enzymes involved in β-oxidation (e.g. acyl-CoA oxidase and carnitine palmitoyl transferase 1 [117]). The NTP concluded that PPARα appears to play a role in several immune effects of PFOA in mice, including decreased spleen and thymus weight, reduced spleen and thymus cellularity, and mitogen-induced lymphoproliferation at high doses (30-40 mg/kg PFOA) [8]. However, many immune effects of both PFOA and PFOS - particularly the suppression of the antibody response in mice at lower doses (3.75 mg/kg PFOA) - are partially or wholly independent of PPARα [67] as is also demonstrated in PPARα knockout mice [68]. Human hepatic PPARα expression is roughly one-tenth that of rodents [118], furthermore e.g. PFOA activates human PPARα with less potency than mouse PPARα [119]. It has to be noted, that such comparative calculations have not been performed for different cell types or life stages and that this fact does not rule out PPARα-induced in immune modulating effects of PFAS in humans.

PPARβ/δ influences cell proliferation, glucose metabolism and inflammation [116]. Its engagement increases the expression of pyruvate dehydrogenase kinase-4 (PDK4) and carnitine palmitoyl transferase 1A (CPT1A), which in turn increases fatty acid oxidation [117]. An association between increased risk of common cold and PPARβ/δ expression in human cord blood has been observed [120]. While studies reported that PFAS such as GenX [121] as well as PFOS and chlorinated polyfluorinated ether sulfonates (Cl-PFAES) [122] can activate PPARβ/δ, downstream molecular events affecting the immune system remain unexplained.

PPAR𝛾 has a wide variety of biological functions, including the regulation of fatty acid synthesis and storage, promotion of adipogenesis, glucose metabolism, and inhibiting inflammatory signalling through NF-κB [123, 124]. PPARγ acts as a transcription factor for genes that contain PPAR response elements in their promoters, including cyclooxygenase-2 (COX-2) [125, 126]. The expression of PPAR𝛾 in B cells is of importance during both the primary and secondary immune response [127]. Furthermore, it is a master regulator for mast cells thereby playing an important role in allergic inflammation [128]. It can increase the adiponectin concentration and expression of glucose transporters, such as GLUT1 and GLUT4 [117], which affects the glycolytic metabolism and cellular metabolism of T cells, respectively [129].

All PPAR isoforms are often co-expressed in developing tissues and organs (e.g., placenta) and the relative levels vary between cell types [130]. Inappropriate activation (i.e., increase or decrease) of one or more PPAR isoforms during critical stages of development could influence the healthy development of a child. The placenta for example has various functions including foetal protection against the maternal immune system and the synthesis of various neurotransmitters and hormones [131]. Bogacka and co-workers [132] discussed that PPARγ-dependent inhibition of various cytokines (e.g. IL-6, IL-1β and TNFα) in the human placenta may influence the immune response and immunotolerance but further studies on this field are required.

##### Modulation of osteoimmunology (via PPARy)

Bone is potentially a significant target tissue of PFAS toxicity [20, 133–135]; six relevant studies were identified for this section. Pérez et al. [136] detected 12 perfluoro alkyl acids (PFAA, including PFCAs and PFSA with a carbon-chain length between C4 and C16) in human rib bone samples (*n =* 20) in Spain. PFOA and PFOS were present in all (*n =* 18) and PFNA, PFDA, PFUnDA and PFHxS in some human bone samples investigated by Koskela et al. [133]. Further, Bodganska et al. [134] found PFBS in the bone marrow of mice when they were orally exposed to PFBS for 5 days.

It is known that osteoclasts and osteoblasts express PPARγ [137] and there are indications that osteoclasts have a major influence on the modulation of immune responses towards immune suppression [138]. Osteoclasts are primarily known for their classical bone resorption activity but are rarely considered as possessing immune functions. However, they have been shown to be involved with immune regulation in the bone marrow [138]. According to Madel et al. [138] osteoclasts have the capacity to activate T-cell responses and modulate T-cell activation, and they produce various cytokines (IL-1β, IL-6, IL-10, TGF-β and TNFα) that affect immune responses as well. PPARγ potentially plays an important role in the production of those cytokines [138], but it is speculative to which extent PFAS may influence immunological functions via this pathway.

Another study [139] reported that the activation of PPARγ in bone marrow suppresses osteoblast and bone formation, and promotes adipocyte differentiation. Continuous activation of PPARγ via its agonists can promote adipogenesis and fatty-acid storage [140], and possibly initiate abnormal bone cell development. An imbalance of osteoclasts and osteoblasts (“bone marrow failure”) can contribute to immune deficiencies and increase the risk of infections [141, 142]. Since the bone marrow is a primary hematopoietic and immune-regulatory organ that probably is exposed to a large variety of PFAS, we hypothesise that PFAS may influence the immune system at least partially via osteoclasts and osteoblasts imbalances in a PPARγ dependent manner. However, since current observations are rather inconclusive, further research on the potential influence of PFAS on bone health and its association with immune responses is recommended.

##### Impact on fatty acid metabolism and secondary effect on the immune system

PFOA and PFOS may indirectly affect the immune system by interfering with lipid metabolism (reviewed by Liang et al. [20]). The authors state that PFOA causes lipid metabolism disorders at least partly via the PPARα pathway, while the mechanism of PFOS-induced interference is not clear.

As mentioned above, there are indications that various PFAS can activate all three PPAR isoforms (see Table 5 in Appendix), which are implicated in regulation of lipid metabolism and/or fatty acid (FA) synthesis and oxidation. The synthesis vs. oxidation of FA has been related to differences in the immune response. FA synthesis is implicated in inflammatory and effector T-cell (Th1, Th2, Th17) responses, while FA oxidation is involved in anti-inflammatory (M2 macrophages), tolerogenic, and CD8+ memory T-cell responses [143]. This difference is also seen at the level of proinflammatory vs. immunosuppressive cytokines. It might therefore be suggested that the effects of PFAS on FA metabolism may affect pro- vs. anti-inflammatory, and effector vs. regulatory T-cell responses.

A link between FA synthesis and immune function is also shown in the work of Wen et al. [144], who found that the saturated FA palmitate induces NLRP3 (NOD-, LRR- and pyrin domain-containing protein 3) inflammasome activation. This results in the production of the pro-inflammatory cytokines IL-1β and IL-18, and in case of chronic activation potentially can be linked to the development of diseases such as type-2 diabetes. Further, adipocyte secretion may affect immune responses, and it is therefore of interest that developmental PFAS exposures seem to be associated with changes in serum-adipokine concentrations [145].

##### Conclusion on potential PPAR modulation

Many studies have demonstrated that PFAS have agonistic effects on human PPARα, PPARβ/δ and PPARγ (see Table 5 in Appendix) and that PPARα and PPARγ are more responsive to PFAS exposure compared to PPARβ/δ which shows weak activity in response to PFAS exposure [146]. An inverse U-shaped relationship between the carbon chain-length of perfluorocarboxylic acids (PFCAs) and the PPARγ transcriptional activity was observed by Li et al. and Zhang et al. [147, 148]. However, study outcomes are inconsistent depending on the cell type and model used.

While PFAS may influence the immune system at least partially via PPARs, the exact mechanisms remain largely unclear. Possible mechanisms include modulation of downstream signalling, such as NF-κB, impact on bone marrow (via PPARγ), and modulation of FA metabolism by all three isoforms and thereby causing a secondary effect on the immune system by tipping it towards inflammation or a suppressed immune response. However, the role of PPARs in the reduced vaccination response in children caused by PFAS is not clear. According to AbdelMassih and co-workers, PPAR agonists (e.g. thiazolidinedione) even have the potential to improve immune responses after vaccination [127]. The response of the immune system after PPAR activation could be either positive or negative, depending on the PPAR agonist type and its concentration. Furthermore, crosstalk from other (nuclear) receptors and variations in downstream signalling add to the complexity of the mechanisms involved.

#### Modulation of calcium signalling

Calcium (Ca^2+^) signalling, spatial and temporal fluctuation of intracellular Ca^2+^ levels, plays a major role in regulating cell functions including innate and adaptive immune responses. In lymphocytes, increases in cytosolic and organellar Ca^2+^ concentrations control crucial effector functions, such as metabolism, proliferation, differentiation, antibody and cytokine secretion and cytotoxicity. Therefore, Ca^2+^ is of paramount importance to immunity, and altered Ca^2+^ regulation in immune relevant cells leads to various autoimmune, inflammatory and immunodeficiency syndromes [149–151]. Immune cell types like macrophages, neutrophils, NK cells, dendritic cells and mast cells are dependent on tightly controlled calcium signalling for their activation and effector functions like degranulation, cytokine release, phagocytosis, cytotoxicity, ROS production and inflammasome activation [152].

Disturbance of Ca^2+^ homeostasis is well known for environmental immunotoxic pollutants like dioxins (such as 2,3,7,8-Tetrachlorodibenzo-p-dioxin, TCDD) or polycyclic aromatic hydrocarbons (PAHs) [153]. The immunosuppressive PAHs benzo[*a*]pyrene (BaP) and dimethylbenz[a]anthracene increased intracellular calcium levels in human lymphocytes and monocytes [154, 155]. Proposed mechanisms included the inhibition of the sarcoplasmic reticulum Ca^2+^-ATPase (SERCA) activity [154], interaction with the ryanodine receptor (RyR1) and protein tyrosine kinases activation mediated by BaP metabolites [155, 156]. After in vitro TCDD exposure activation of the calcium/calmodulin signalling pathway and an increase in intracellular calcium led to mitochondrial dysfunction associated with apoptosis in a human lymphoblastic T-cell line model [157]. Depending on the magnitude and duration of changes in Ca^2+^ signalling, various consequences are possible, such as suppression of humoral and cell-mediated immunity, apoptosis, immune enhancement or proliferation.

##### Experimental studies on modulation of calcium signalling by PFAS in immune cells

A total of six relevant experimental studies was identified, dealing with modulation of calcium homeostasis in immune cells. Of these studies, four originated from our literature research (see Methods) in addition to the discussion of two studies already published before 2018. Mechanistic information on the disruption of calcium signalling in immune cells by PFAS is limited and mainly deal with innate immunity (see Table 6 in Appendix). A large body of evidence for the modulation of calcium homeostasis by PFAS in a variety of cell types (besides immune cells) and models exist (data not shown).

Table 6 in Appendix lists experimental studies showing modulation of calcium homeostasis by PFAS in immune relevant cells.

Wang and co-workers [158] demonstrated, that PFOS increases cytosolic Ca^2 + −^ in human and mouse macrophages and activates the AIM2 (absent in melanoma 2) inflammasome in a process involving mitochondrial DNA release through the Ca^2+^ dependent protein kinase C (PKC)-NF-κB/ c-Jun N-terminal kinase (JNK)-BAX/BAK axis. This process results in the production of multiple proinflammatory cytokines, leading to endoplasmic reticulum (ER)-stress, cellular injury and tissue inflammation. Four in vitro studies [111, 128, 159, 160] investigated mast cell-mediated allergic inflammation as well as allergy/anaphylaxis-models and concluded that the respective PFAS tested aggravated IgE-dependent allergic symptoms. They all show an increase in intracellular Ca^2+^ levels in mast-like cells (mostly rat, but also human cells) after treatment with PFOA [128, 159], PFOS [160], PFDA and PFUnDA, but not PFNA and PFHpA [111]. The authors of the latter study therefore conclude that carbon chain length of PFAS may serve as a factor in allergic inflammation. A key step for mast cell activation is antigen-mediated cross-linking of IgE via the high-affinity IgE receptor (FcɛRI) that can trigger calcium mobilisation by two different modes: store-operated Ca^2+^ entry (SOCE) and non-SOCE (e.g. L-type Ca^2+^ channels) [161, 162]. In all four studies, as a consequence of modulation in Ca^2+^-levels, increased levels of histamine, ß-hexosaminidase and augmented mast cell degranulation were observed.

In human bone mesenchymal stem cells, genes related to calcium signalling were upregulated for 6:2 chlorinated polyfluorinated ether sulfonate (F-53B), PFOS, PFHxS and PFOA. Subsequent calcium changes were enhanced for F-53B with lower effective concentrations and a more prolonged induction compared to PFOS and PFHxS [163].

##### Ca^2+^-related mechanisms in other cell types

An increase in cytosolic Ca^2+^ seemed to be induced by PFAS in most study models using different cell types. Ca^2+^ influx and efflux can occur across the plasma membrane and within the cytosol across the ER, mitochondria or lysosomes and is mediated by specific channels and transporters that are part of complex signal transduction cascades [149, 150].

A body of experimental studies show possible Ca^2+^ mediated mechanisms for (developmental) neurotoxicity including interaction with different Ca^2+^-sensitive receptors such as N-methyl-D-aspartate, L-type gated voltage calcium channels [164–167], and it should be investigated further if this could play a role in (developmental) immunotoxicity.

We aimed to bridge mechanistic information observed from other cell types to immune cells, in order to help elucidate additional possible targets of PFAS and immune system related consequences. Depletion of Ca^2+^ stores in the ER results in SOCE and subsequent activation of plasma membrane calcium release-activated calcium channel (CRAC). This mechanism is relevant for the activation of immune cells such as T- and B- cells, NK cells and mast cells [151] and thus important for immunity to infections or antibody production [150, 152]. For example, in T-cells activation of the T-cell receptor leads to Ca^2+^ release from the ER stores by inositol 1,4,5-trisphosphate receptors (IP_3_R) channels and subsequent activation of the calcineurin and the nuclear factor of activated T-cells (NFAT-) pathway [149]. Mitochondria take up and extrude Ca^2+^ for proper T-cell responses after T cell receptor ligation [150].

L-type voltage-gated channels are also expressed in T-cells and are required for fine-tuning of T-cell activation, cytokine production and Th2 function in asthma [149, 152]. RyRs channels in the ER release Ca^2+^ after activation with disturbances leading to possible inhibition of the formation of the immune synapse in T cells amongst others [149].

While several mechanisms observed with experimental (in vitro) models are thus likely relevant for immune cells, experimental evidence on potential consequences of increases in cytosolic Ca^2+^ caused by PFAS exposure includes: i) the activation of downstream kinases, such as PKC, leading to the activation of NF-κB, JNK or p38-MAPK signalling, ii) oxidative stress and generation of ROS, iii) apoptosis, iv) induction of degranulation of mast cells and v) reduced antibody production (T- and B-cells). However, the mechanistic link between reduced antibody production and altered calcium signalling is not established yet.

In conclusion, PFAS show the ability to alter calcium homeostasis in immune cells as evidenced by increases in intracellular calcium concentrations in several in vitro models.

Additional investigations on other immune cell types (e.g. T-cells and B-cells) and molecular targets including Ca^2+^- channels and associated factors/proteins for calcium signalling would be needed to gain insight into the exact mode of action of the observed calcium imbalance due to PFAS exposure. The complexity and network of calcium signalling with vital cell processes including immune function makes it challenging to assign adverse outcomes to calcium signalling disruption.

### Induction of oxidative stress and potential consequences for immune health

PFAS have been shown to induce oxidative stress, which occurs when the amount of oxidants such as reactive oxygen species (ROS) in a cell exceeds its antioxidant capacity. Ten relevant studies, describing PFAS-induced oxidative stress, have been identified from our literature research (see Methods). The formation of ROS by PFAS has been reviewed in a recent article by Gundacker and co-workers [168]. The most common source of ROS formation intracellularly are mitochondria, which are also an immediate target of ROS. This may have pathological consequences, e.g. oxidative damage of mitochondrial DNA may trigger cell apoptosis by inducing mitochondrial stress and downstream signalling [169]. Mitochondria play a key role in the regulation of the immune system [169, 170]. For example, T cell activation is dependent on oxidative phosphorylation and ROS production, while activated T cells can use either oxidative phosphorylation or glycolysis for proliferation [169]. Mitochondria are essential for the regulation of metabolism in different types of immune cells through glucose oxidation as well as the biosynthesis of fatty acids, amino acids and hormones – this is critical for their survival, proliferation and activation [170]. Furthermore, mitochondria are capable of activating innate immune responses, such as the activation of NF-kB signalling pathways and the inflammasome [169]. Mitochondrial ROS is one of the mitochondrion-derived molecules that contribute to activation of the NLRP3 inflammasome, which has been shown to lead to secretion of proinflammatory cytokines [170, 171]. Overall, mitochondrial ROS is considered a regulator of the immune system, as it provides signals that lead to cell state/fate determination [170].

Members of the PFAS family have been shown to induce ROS formation in human lymphocytes (PFOS) [172], in murine astrocytes and mouse primary hepatocytes (PFOS and PFOA) [173, 174]. Furthermore, PFAS have been shown in vivo and in vitro to induce oxidative stress by affecting Nrf2 and its target genes. Oral PFOS exposure of male mice was shown to result in a substantial suppression of hepatic protein levels of Nrf2, which in turn led to the production of malondialdehyde (MDA), suppressed the activity of superoxide dismutase (SOD) and reduced glutathione (GSH) content in liver homogenates [175]. Supplementing the PFOS treatment with naringin, a naturally occurring flavonoid glycoside with antioxidant properties, increased the Nrf2 expression and alleviated the oxidative stress response. Other in vitro studies using various cell types have reported declines in GSH content together with increases of MDA content upon exposure to different PFAS, such as PFOS, PFOA and PFNA [172, 173, 176, 177]; study details for references [172–177] are described in Table 7 in Appendix. Further support for the involvement of Nrf2 was obtained from murine *nrf2*^*−/−*^ astrocytes, which showed in response to PFOS or PFOA treatment a significant decrease in GSH/GSSG ratio as compared to their wildtype counterparts [173]. One study using mouse primary hepatocytes reported the opposite effect, i.e. an increase in GSH content upon exposure to PFOS or PFOA, which the authors considered as adaptation to oxidative stress, leading to suppressed GSH content and detoxification of oxidized GSSG to GSH [174]. Additionally, the majority of these in vitro studies reported alterations of the major enzymatic antioxidants SOD, catalase (CAT), and glutathione peroxidase (GPx) [173, 174, 176, 177]. Adverse effects on mitochondria were observed through a decrease in mitochondrial membrane potential [172] and substantial changes in morphology [173]. Given that these PFAS-induced effects were observed in different cell types, it is likely that similar effects may occur in cell types relevant to the immune system.

Additionally, PFAS have been reported to alter calcium signalling and calcium homeostasis (see Modulation of key cell populations - animal studies section). This also impacts mitochondria, which play a major role in Ca^2+^ signalling throughout the cell. For example, they enhance bioenergetics necessary for T-cell activation and proliferation via uptake of cytosolic Ca^2+^ [149]. By disturbing the Ca^2+^ modulating function of mitochondria, PFAS may cause mitochondrial Ca^2+^ overload and increase mitochondrial ROS, which further contributes to an affected immune response. In conclusion, PFAS may affect the immune response by inducing oxidative stress and mitochondrial dysfunction.

### Modulation of NK cell activity

NK cells are innate lymphoid cells that play an important role in antiviral responses and tumour defence. NK cell activity is a measure of non-specific immunity and a commonly used parameter in immunotoxicity studies and a potential mediator of PFAS-associated suppression of disease resistance. Eleven experimental studies are discussed in this section; of these nine have been published before 2018 and two originate from our literature research (see Methods). NK cell counts, and activity has been examined in several mouse studies, mostly in response to PFOS exposure. Several of these studies show a reduction in NK cell numbers and inhibition of NK cell cytolytic activity in PFAS exposed adult animals [64, 71, 72] and after prenatal exposure of mice to PFOS (GD 1-17) or PFOA (GD 1-13) [74, 178]. However, in two mouse studies including low exposures (from 0.166 μg PFOS/kg bw/day in a 28 day study and from 8.33 μg/kg bw/day in a 60 day study), an increase in NK cells activity [70, 72] was reported in males at concentrations up to 5 mg/kg total administered dose but not associated with reduced splenic and thymic cellularity. For study designs of the following references: [62, 68–70, 72], please see also Table 3 in Appendix; study design of reference [176] is described in Table 7 in Appendix. In an in vitro study with 24 hour exposure of PBMCs to either PFOS and PFOA [179], inhibition of NK cell activity by PFOS, but not of PFOA was reported.

In addition to their cytolytic activity, NK cells secrete several cytokines, that can modulate innate and adaptive immune responses [180], however possible effects of PFAS on such properties are not known. The NTP concluded in 2016 that the evidence on NK cell modulations by PFOA was weak due to limited studies, but there was moderate confidence that exposure to PFOS is associated with changes in NK cell activity in animals [8]. Few new data on PFAS-associated changes in NK cell number and activity were found in our literature review. However, a recent study with gestational exposure (GD 1-13) to PFOA, by Jiang and co-workers [178], showed a reduced number of uterine NK cells at the maternal-foetal interface; for study details see Table 7 in Appendix. In conclusion, available data indicate that PFOS and likely also PFOA cause a decrease in NK cell activity and number. Based on the study by Dong and co-workers [72], one could speculate, that a non-monotonic dose response is possible. However, further characterisation of the NK cell changes in response to human-relevant PFAS exposure levels and its potential contribution to changes in infectious disease risk are needed.

### Immunoenhancement

Immunoenhancement (immuno-stimulation) can broadly be defined as inappropriate activation of the immune system and may result in hypersensitivity responses such as allergy or asthma or as autoimmune reactions where the immune system responds to self-antigens [14, 181].

#### Hypersensitivity

As detailed above in section, “Knowledge on immunomodulatory effects of PFAS from epidemiological studies”, data from prospective studies appear inconclusive concerning an association between PFAS exposures and asthma. However, several cross-sectional studies report increased risk of asthma, and it is hypothesised that PFAS may exacerbate existing asthma, but have a minor role in disease development. No conclusion could be drawn regarding potential effects of PFAS exposures on lung function or other hypersensitivity-related health outcomes.

The NTP concluded in 2016 [8], that there is moderate confidence that exposure to PFOA is associated with increased hypersensitivity responses based on the available animal studies [128, 182, 183]; see NTP review [8] for discussion of study details. Mechanistic data for PFOA-associated hypersensitivity suggested the response is IgE-mediated and may involve stimulation of mast cells, but a clear pattern of effects on inflammatory cytokines or the role of NF-κB at relevant PFOA concentrations had not been established. For PFOS, there were few experimental studies available [73, 182] and due to inconsistent results from animal and human studies, the evidence for an association with hypersensitivity reactions with PFOS exposure was ranked as low at that time [8]. In addition to the evidence reviewed by the NTP [8], our literature review (see Methods) identified five more recent (2018 and newer) studies. Several in vivo studies address the possible effects of PFAS on hypersensitivity reactions [111, 160, 184–187]. For study designs, please see Table 6 in Appendix [111, 160] and Table 7 in Appendix [184–187]. These studies provide additional support that high to moderate exposures to PFOA and PFOS may aggravate allergic lung responses in ovalbumin (OVA) sensitised mice [111, 160, 184, 185]. These studies further suggest that PFAS may increase serum IgE and OVA-specific IgE, and change cytokine production towards a Th2-dominated response in mice [160, 184, 185].

In the study by Wang et al. [187], pre-treatment of mice with intranasal administered PFOS was shown to reduce early life allergic asthma responses to house dust mites in a mouse model of allergic asthma and dampen the Th2 response. The authors report that PFOS bind to and inactivate Der p1, the major immuno-active component of the house dust mite as well as to lipopolysaccharides (LPS) and thus alters the in vivo responses to these molecules. In this study, PFOS also inhibited the response to *Pseudomonas aeruginosa* infection. The study underlines that several modes of action may contribute to how PFAS may modulate early life antigen responses.

Several in vitro experiments have measured the release of hypersensitivity mediators, like histamine and β-hexosaminidase, and cytokines in cultured cells after PFOA and PFOS exposure [111, 160, 186] and in sensitised mast cells [111, 160] exposed to PFOS and long-chained PFCAs. These studies support that, at least at higher concentrations, PFAS may exacerbate airway hypersensitivity reactions. These studies are further detailed above in section “*Modulation of calcium signalling*” and in Tables 6 and 7 in Appendix.

#### Autoimmune diseases

Autoimmune disease and related effects are the result of immune responses against self-molecules [14]. The NTP concluded that the evidence from both human and animal studies translate into inadequate level of evidence for an association with PFOS exposure. There was weak evidence from human studies, that PFOA exposure was linked to ulcerative colitis [188, 189], and inconsistent findings for rheumatoid arthritis [8]. Our literature review (see Methods) identified three more recent (2018 and newer) relevant studies. In a recent update from former C8 Science Panel members and collaborators, it was concluded that there is evidence for an association between PFOA exposure and ulcerative colitis (UC), but not for other auto-immune diseases [190]. This was also the conclusion of the ATSDR report [9]. Since UC is only restricted to the colon/rectum (and no association with Crohn’s disease was observed), it was discussed this may involve effects of PFOA on bacterial exposure unique to the lower GI tract as well as inflammation-mediated mechanisms. However, no positive association between PFAS and UC was observed in the most recent study [191].

Experimental animal data for autoimmunity are scarce. Using an experimental mouse model of autoimmune diabetes, Bodin and co-workers showed that PFUnDA exposure of female mice from conception and up to 30 weeks of age exacerbated pancreatic insulitis development, a potential early marker for type 1 diabetes (T1D), but did not accelerate diabetes development [192]. A recent scoping review included three epidemiological studies examining associations between different PFAS and T1D, but no clear trends could be identified [193].

In conclusion, the evidence that PFAS contribute to risk of autoimmune diseases is currently weak.

## Discussion

**Fig. 2:**
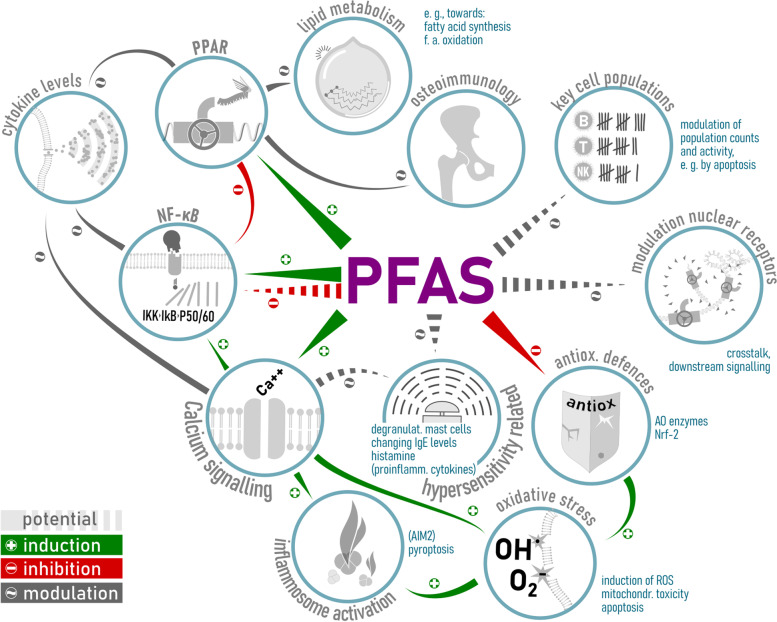
Mechanistic considerations of modulation of (developmental) immune functions by PFAS. Figure Legend: Relevant mechanisms that may underlie the observed effects on the (developmental) immune system are: i) modulation of cell signalling and nuclear receptors, such as NF-κB, PPARs and others; downstream signalling might vary due to receptor crosstalk; ii) alteration of calcium signalling and calcium homoeostasis in immune cells (having an impact on oxidative stress, inflammasome activation, nuclear receptors such as NF-κB, cytokine levels and degranulation of mast cells); iii) modulation of key cell populations necessary for an antibody response; iv) modulation of NK cells; v) modulation of mast cells and IgE influencing hypersensitivity; vi) oxidative stress and vii) impact on fatty acid metabolism and secondary effects on the immune system. Green arrows indicate induction, red arrows inhibition, grey arrows modulation in either direction and dashed lines potential modulation (with a higher degree of uncertainty)

This review aimed to collect available information and describe hallmarks of the molecular mechanisms leading to PFAS-induced immunotoxicity. Figure 2 provides an overview of the mechanisms discussed within this article. A full understanding of the mechanisms has not yet been achieved for various reasons, including the use of different methods and models to investigate different types of immune responses for single members of the PFAS group.

In addition to their unique chemical and physical properties, PFAS exert different as well as partly overlapping types of immunotoxicity, and amongst other factors, crosstalk of (nuclear) receptors and variation in downstream signalling add to the complexity.

The immunotoxicity of PFAS have been in focus recently and several reviews have been published [11, 20, 39, 194]. While Antoniou and co-workers conclude that more evidence would be needed to select immunomodulation as a critical endpoint for human PFAS risk assessment [194], other reviews postulate strong evidence for PFAS exposure on diminished childhood antibody vaccination response [11, 39]. The data presented in this review strengthens the evidence that PFAS indeed do show immunomodulatory activities in vitro, in animals and in humans. The associations between PFAS and reduced vaccination specific antibody responses in children is strong and the evidence is strengthened also with regard to an increased risk of common infectious disease.

### Data gaps and recommendations

#### Mechanism of action of immunotoxicity of PFAS

Even though a large body of data on some PFAS exists, further research to address data gaps is needed. As Fragki and co-workers [109] point out, human-relevant test systems would be ideal to obtain more insight into the mechanistic pathways for immunotoxicity pertinent to humans. These studies should be designed with a careful consideration of appropriate dosing and toxicokinetics, so as to enable biologically plausible quantitative extrapolations.

Emphasis should be placed on describing AOPs (Adverse Outcome Pathways) involved in PFAS-induced immunotoxicity, given the (very) limited number available in the AOP Wiki (aopwiki.org) and the scientific literature. This would greatly facilitate the identification of appropriate new approach methodologies (NAMs) to generate relevant mechanistic information for substances without the need to perform additional animal experiments, and to facilitate read-across to chemicals with more data-rich (in vivo) toxicity databases. PFOS and PFOA are the most studiedPFAS, but their mode of action has not been fully identified, and available mechanistic information on other PFAS is limited. To this respect, the recent efforts under the OECD Working Party of the National Coordinators of the Test Guidelines Programme (WNT) [195], where an Ad hoc expert group was established to develop a detailed review paper on the application and interpretation of in vitro immunotoxicity assays and definition of a tiered approach to testing and assessment, is a promising step in this direction.

Similar to the AOP framework, key characteristics (KCs), that describe properties of agents or exposures that confer toxicological hazards, can be used as an organising principle for research supporting the evaluation of compounds of concern. Very recently, during the submission phase of this paper, a committee of 18 experts with diverse areas of expertise published 10 key characteristics (KCs) of immunotoxic agents: 1) covalent binding to proteins to form novel antigens, 2) affecting antigen processing and presentation, 3) alteration of immune cell signalling, 4) alteration of immune cell proliferation, 5) modification of cellular differentiation, 6) alteration of immune cell-cell communication, 7) alteration of effector function of specific cell types, 8) alteration of immune cell trafficking, 9) alteration of cell death processes, and 10) breaking down immune tolerance [196]. Various PFAS, not only PFOA and PFOS, affect multiple aspects of the immune system and therefore very likely show several of these 10 KCs of immunotoxic agents: e.g., KC no. 2) ‘affecting antigen processing and presentation’ could be indicated by the findings with TDAR assays with PFOA and PFOS (see Repeated dose toxicity and immunotoxicity studies in animals section); KC no 3) ‘alteration of immune cell signalling’ via the modulation of nuclear receptors (such as NF-κB and PPARs; see Modulation of NF-κB regulated gene transactivation and Involvement of PPARs sections) or Modulation of calcium signalling); Section 4.3; KC no. 9) ‘alteration of cell death processes’ via induction of oxidative stress, to name a few. Further investigation of the KCs of immunotoxicity of PFAS is highly recommended.

This could be a topic of priority for the recently launched Horizon Europe Partnership for the Assessment of Risks from Chemicals (PARC; https://www.anses.fr/en/content/european-partnership-assessment-risks-chemicals-parc), which will likely make a significant contribution to this area. Immunotoxicity has been designated as one of the toxicological effects for which (networks of) AOPs and IATAs (Integrated Approaches to Testing and Assessment) will be generated and PFAS have been chosen as a priority substance group.

Additional investigations on other immune cell types (e.g., T-cells and B-cells) and molecular targets including Ca^2+^- channels and associated factors/proteins for calcium signalling would be needed to gain better insight into the exact mode of action of the observed calcium imbalance due to PFAS exposure.

Given the complexity and uncertainty regarding the mechanisms underlying immunotoxicity in the case of PFAS, NAMs must be carefully applied. Promising examples are the use of mechanistic computational platforms, such as the Universal Immune System Simulator, UISS-TOX as described by Pappalardo and co-workers and in vitro high throughput platforms (as described by Naidenko et al. [197]. Assumptions for the immunotoxicity mode of action of PFAS had to be taken by Pappalardo and co-workers for the UISS-TOX platform [198] that might not be fully supported by currently available data. Furthermore, PFAS show the limitations of the currently available high-throughput assays for immunotoxicity screening in the U.S. EPA ToxCast Program, as Naidenko and co-workers point out. The authors state that the existing assays likely do not capture the full extent of the possible mechanisms of immunotoxicity, especially in different immune cell subpopulations [197].

On the other hand, in terms of risk assessment and management, the research already available on legacy PFAS may be useful to elucidate the susceptibilities of the immune system and its critical windows during development that may also relate to similar toxicity from other environmental chemicals.

#### Current guidelines and gaps in immunotoxicity testing and regulatory risk assessment

For PFAS, as for many other agents, the developing immune system may be more sensitive than the adult immune system. Identification of the critical windows of exposure related to adverse effects to the immune system is essential.

A comprehensive risk assessment requires that all types of immunotoxicity be addressed, as clearly indicated in the guidance for immunotoxicity risk assessment for chemicals [14]. The goal is to detect chemically induced immune dysfunction with an impact on health risk, which is best achieved by testing host-resistance to a foreign challenge in functional assays. However, the current data requirements requested as part of chemical legislative frameworks such as the REACH Regulation do not align with the demands for assessing all aspects of the (developing) immune system. For instance, the TDAR assay is only requested optionally under REACH as part of the extended-one generation repeated-dose toxicity (EOGRT) study (OECD TG 443), based on positive immune- or endocrine-related findings that are (amongst others) observed in repeated-dose toxicity studies. Thus, it cannot be excluded that many of the substances that will cause (developmental) immune effects other than skin sensitisation will currently remain unnoticed. Hence, efforts are urgently needed to lower the threshold of immunotoxicity testing in standard regulatory evaluations of e.g., industrial chemicals, biocides or pesticides.

#### Additional data gaps

Human studies may have underestimated exposure during early development of the immune system and efforts should be undertaken to better assess early life exposures. Due to the sensitivity of the developing immune system, importance should be given to the measurement of PFAS serum levels during pregnancy and in umbilical cord blood.

An important question also might be how PFAS exposure might affect potential susceptibility and severity of viral illness, including but not limited to COVID-19. Studies providing evidence that PFAS may alter COVID-19 risk via epigenetically-regulated immune pathways have been summarised by Bulka and co-workers [199]. Epigenetic immune modifications (such as changes in DNA methylation) may provide mechanistic insights into the decreased antibody response observed after vaccination and further research is necessary to investigate this relationship.

According to the recommendations by EFSA [10], more longitudinal epidemiological studies are needed on human endpoints, in particular prospective vaccination studies covering other types of vaccines, different populations, as well as more studies on other immune outcomes in humans, including the risk of infections.

Due to their high contribution to the PFAS levels observed in human serum, EFSA points out the need for more experimental studies with PFNA and PFHxS on the immune system. This scope might be widened to frequently found PFAS-replacement products, as detected in bodily fluids of the general population in the U.S. [200], and China [201], such as 9-chlorohexadecafluoro-3-oxanone-1-sulfonic acid (9Cl-PF3ONS also known as 6:2 Cl-PFESA, trade name “F-35B”), perfluoro-3,5,7,9-tetraoxadecanoic acid (PFO4DA) or perfluoro-3,5,7,9,11-pentaoxadodecanoic acid (PFO5DoA), amongst others.

PFAS grouping according to their toxicological profile and the development of relative potency would greatly assist risk assessment approaches for the evaluation of PFAS mixtures and risk management. In an attempt to shed more light on the differences in the immunotoxic potential and potency between PFAS, animal experiments were evaluated in an upcoming publication by Bil and co-workers, with the purpose of deriving so-called Relative Potency Factors (RPFs) for immune effects [202]. The RPF methodology allows performing risk assessments for combined exposures to multiple compounds, which is of relevance to assessing the risk of PFAS mixtures [203]. The authors successfully derived internal RPFs for decreased thymus weight, spleen weight, and globulin in rodents, but the available dose-response information for blood cell counts was insufficient for the derivation of RPFs. The results from the studies using internal RPFs indicate that internal RPFs based on liver weight increase as well as the newly derived RPFs based on decreased weight of lymphoid organs are similar [202]. Studying of relative potencies of PFAS using NAMs and accompanying quantitative in vitro-in vivo extrapolation (QIVIVE) methods may support these findings (EFSA tender: [204]).

## Conclusions

Taken together, there is ample evidence illustrating PFAS affect multiple aspects of the immune system, which supports the overall conclusion that not only PFOA and PFOS, but also other members of the PFAS family alter immune functions in humans. The most reported immunotoxic effect in humans is immunosuppression, reflected by reduced vaccine antibody levels and increased risk of common infectious diseases. At present, a clear picture of effects on immune cell phenotypes could not be derived from epidemiological studies.

Mechanistic in vitro studies provide further evidence for PFAS-induced immunotoxicity, demonstrated by modulation of nuclear receptors (e.g., NF-κB, PPARs), Ca^2+^-signalling, as well as modulation of oxidative stress and cytokine levels. In in vivo studies, a robust pattern of PFOA- and PFOS- associated immunosuppression has been observed with the TDAR assay, with less data available for other PFAS members. Experimental animal studies underline the resting immune system as a relevant target for PFAS-induced toxicity. Animal studies show further, that some PFAS, including PFOS and PFOA, can reduce splenic and thymic cellularity and levels of circulating WBC.

Timing of exposure is critical, because the developing immune system is especially vulnerable to toxic insults, resulting in a higher risk of immune effects in infants and children. However, the current data requirements requested as part of chemical legislative frameworks such as REACH do not sufficiently align with the demands for assessing all aspects of the (developing) immune system.

## Data Availability

Data sharing is not applicable to this article as no datasets were generated or analysed. Studies cited in this review are publicly available (see List of References).

## Appendix

Tables 1, 2, 3, 4, 5, 6 and 7

**Table 1:** Literature research: databases, search terms and search combinations (see Methods section). Within the frame of this review, this database was used for experimental studies (in vitro and in vivo)

Database	Date	Keyword A	Keyword B	N
Web of Sciences	08.06.2021	PFAS	immune*, TDAR*, TIAR*, TIAR*, SRBC*, IgG*, IgE*, Host infection studies, viral infection, bacterial infection, innate immunity, hypersensitivity, B-cell function, plasma cell function, lymphotoxicity, Lym*, thymus atrophy, thymu*, spleen atrophy, spleen*, thymus cellularity, spleen cellularity, PPAR*	88
Web of Sciences	09.06.2021	PFAS	natural killer cell, cytokine*, IL-1R1*, IL-1Ra*, interleukin*, NF-kB*, macrophage*, mast cells, histamine*, hexosaminidase*, IFN-gamma*, T-cell, reactive oxygen species, mitochondria*, apoptosis*, host infection, inflammasome*, Nrf-2*, Nrf2*, NFAT*, calci*, neopterin*, tryptophan*, glucocorticoid*, JAK3*, p53*, Bax*, superoxiddismutase*, superoxid*, glutathion*, GST*, lipid peroxidation, caspase*, BcL-2*, BcL2*, fatty acid metabolism, innate immunity, hypersensitivity, asthma*, allerg*, respiratory infection, autoimmunity	185
Web of Sciences	23.06.2021	perfluor*, polyfluor*, PFBS*, PFHxS*, PFOS*, PFBA*, PFHxA*, PFOA*, PFNA*, PFDA*, PFUnDA*, PFDoDA*, HFPO-DA*, GenX*	TDAR*, TIAR*, SRBC*, KLH*, HRBC*, “host infection”, T-cell, infect*,	3
PubMed	10.06.2021	PFAS, perfluor*, polyfluor*, PFBS*, PFHxS*, PFOS*, PFBA*, PFHxA*, PFOA*, PFNA*, PFDA*, PFUnDA*, PFDoDA*, HFPO-DA*, GenX*	immune, TDAR, T-cell, TIAR, SRBC, IgG, IgE, “host infection”, “viral infection”, “bacterial infection”, “innate immunity”, hypersensitivity	192
PubMed	11.06.2021	PFAS, perfluor*, polyfluor*, PFBS*, PFHxS*, PFOS*, PFBA*, PFHxA*, PFOA*, PFNA*, PFDA*, PFUnDA*, PFDoDA*, HFPO-DA*, GenX*	B-cell, “plasma cell function”, lymphotoxicity, “thymus atrophy”, “spleen atrophy”, “thymus cellularity”, “spleen cellularity”, PPAR, “natural killer”, cytokines	192
PubMed	12.06.2021	PFAS, perfluor*, polyfluor*, PFBS*, PFHxS*, PFOS*, PFBA*, PFHxA*, PFOA*, PFNA*, PFDA*, PFUnDA*, PFDoDA*, HFPO-DA*, GenX*	cytokines, interleukin, NF-kB, macrophage, “mast cells”, histamine, hexosaminidase, IFN-gamma,	108
PubMed	13.06.2021	PFAS, perfluor*, polyfluor*, PFBS*, PFHxS*, PFOS*, PFBA*, PFHxA*, PFOA*, PFNA*, PFDA*, PFUnDA*, PFDoDA*, HFPO-DA*, GenX*	“reactive oxygen species”, mitochondria, apoptosis, inflammasome, Nrf-2, Nrf2, NFAT, calcium, neopterin, tryptophan, “glucocorticoid receptor”, JAK3, p53, Bax, “superoxid dismutase”, glutathione, “GSH-transferase”, GST,	187
PubMed	14.06.2021	PFAS, perfluor*, polyfluor*, PFBS*, PFHxS*, PFOS*, PFBA*, PFHxA*, PFOA*, PFNA*, PFDA*, PFUnDA*, PFDoDA*, HFPO-DA*, GenX*	Inflammasome, Nrf-2, Nrf2, NFAT, calcium, neopterin, tryptophan, “glucocorticoid receptor”, JAK3, p53, Bax, “superoxid dismutase”, “glutathione”, “GSH-transferase”, GST,	191
PubMed	15.06.2021	PFAS, perfluor*, polyfluor*, PFBS*, PFHxS*, PFOS*, PFBA*, PFHxA*, PFOA*, PFNA*, PFDA*, PFUnDA*, PFDoDA*, HFPO-DA*, GenX*	“lipid peroxidation”, caspase	41
PubMed	17.06.2021	PFAS, perfluor*, polyfluor*, PFBS*, PFHxS*, PFOS*, PFBA*, PFHxA*, PFOA*, PFNA*, PFDA*, PFUnDA*, PFDoDA*, HFPO-DA*, GenX*	BcL-2, BcL2, “fatty acid metabolism”, “innate immunity”, hypersensitivity, asthma	116
PubMed	18.06.2021	PFAS, perfluor*, polyfluor*, PFBS*, PFHxS*, PFOS*, PFBA*, PFHxA*, PFOA*, PFNA*, PFDA*, PFUnDA*, PFDoDA*, HFPO-DA*, GenX*	asthma, allergies, “respiratory infection”, autoimmunity,	160
PubMed	20.06.2021	PFAS, perfluor*, polyfluor*, PFBS*, PFHxS*, PFOS*, PFBA*, PFHxA*, PFOA*, PFNA*, PFDA*, PFUnDA*, PFDoDA*, HFPO-DA*, GenX*	infection	141
Scopus	08/09.07.2021	perfluor*, polyfluor*, PFBS*, PFHxS*, PFOS*, PFBA*, PFHxA*, PFOA*, PFNA*, PFDA*, PFUnDA*, PFDoDA*, HFPO-DA*, GenX*	immune*, TDAR*, TIAR*, SRBC*, KLH*, HRBC*, PPAR*, infect*, T-cell*, antibod*	80

Please see also List of Abbreviations, for description of Table see Methods section

**Table 2:** Literature search criteria for epidemiological studies: performed in August 2021 to cover papers published on PFAS not included in the EFSA opinions (2018, 2020), as well as papers published after July 2019 for the 27 PFASs included in the EFSA 2020 Opinion [10]. The original search included studies with several health outcomes. Thus, for the present review, immunotoxicological outcomes were used as inclusion criteria in the initial manual screening process. The literature search for the immune outcomes in humans was extended to cover the period from August 2021 to January 2022

Search No.	Keywords and combination of search strings	N
1	(((perfluoroalkyl carboxylic or perfluorobutanoic or perfluoropentanoic or perfluorohexanoic or perfluoroheptanoic or perfluorooctanoic or perfluorononanoic or perfluorodecanoic or perfluoroundecanoic or perfluorododecanoic or perfluorotridecanoic or perfluorotetradecanoic or perfluoropentadecanoic or perfluorohexadecanoic or perfluorooctadecanoic) adj acid?) or PFCAs or PFBA or PFPeA or PFHxA or PFHpA or PFOA or PFNAor PFDA or PFUnDA or PFDoDA or PFTrDAor PFTeDA or PFPeDA or PFHxDA or PFODA or ((perfluoroalkane or perfluorobutane or perfluorohexane or perfluorooctane or perfluorodecane or perfluoroalkane) adj sulfonic acid?) or PFSAs or PFBS or PFHxS or PFOS or PFDS or PFSIAs or PFOSI or FASAs or FOSA or EtFASAs or EtFOSA or EtFASEs or EtFOSE or FC-807 or (n:2 adj (“fluorotelomer alcohol?” or FTOHs or “polyfluoroalkyl phosphoric acid ester?”)) or (8:2 adj (FTOH or “fluorotelomer alcohol” or monoPAP or “fluorotelomer phosphate monoester” or diPAP or “fluorotelomer phosphate diester”)) or ((perfluoroalkane or perfluorooctane) adj sulfonamide?) or (N-ethyl adj (perfluoroalkane or perfluorooctane) adj sulfonamidoethanol?) or “perfluoroalkyl phosphate” or “ammonium bis[2-[N-ethyl(heptadecafluorooctane)sulphonylamino]ethyl]phosphate”).tw,kf.	6557
2	limit 1 to yr = “2018 -Current”	2578
3	(TFMS or ((triflurooacetic or Trifluoromethanesulfonic) adj acid?)).tw,kf.	447
4	(PFCAs or PFBA or PFPeA or PFHxA or PFHpA or PFOA or APFO or PFNA or PFDA or PFUnDA or PFDoDA or PFTrDA or PFTeDA or PFPeDA or PFHxDA or PFHpDA or PFODA or perfluoroalkyl carboxylate? or ((“perfluoroalkyl carboxylic” or perfluorobutanoic or perfluorobutyric or perfluoropentanoic or perfluorohexanoic or perfluoroheptanoic or perfluorooctanoic or perfluorononanoic or perfluorodecanoic or perfluoroundecanoic or perfluorododecanoic or perfluorotridecanoic or perfluorotetradecanoic or perfluoropentadecanoic or perfluorohexadecanoic or perfluroheptadecanoic or perfluorooctadecanoic) adj acid?)).tw,kf.	5029
5	(PFSAs or PFBS or PFPeS or PFHxS or PFHpS or PFOS or PFNS or PFDS or perfluoroalkane sulfonate? or ((perfluoroalkane or perfluorobutane or perfluoropentane or perfluorohexane or perfluoroheptane or perfluoroheptane or perfluorooctane or perfluorononane or perfluorodecane) adj “sulfonic acid?”)).tw,kf.	4366
6	(PFSIAs or PFOSI or ((perfluoroalkane or perfluorooctane) adj “sulfinic acid?”)).tw,kf.	6
7	(PFECAs or HFPO-DA or GenX or DONA or ADONA or HFPO dimer acid fluoride or HFPO-TA or PFO4DA or PFO5DoDA or EEA-NH4 or mv31 K+ or F-DIOX or ((“perfluoroalkyl ether carboxylic” or “hexafluoropropylene oxide dimer” or “dodecafluoro-3H-4,8-dioxanonanoic” or “perfluoro-3,5,7,9-butaoxadecanoic” or “perfluoro-3,5,7,9,11-pentaoxadodecanoic”) adj acid?) or “Hydro-Eve” or “ammonium difluoro[1,1,2,2-tetrafluoro-2-(pentafluoroethoxy)ethoxy]acetate” or “potassium 2-(3-trifluoromethoxy-1,1,2,2,3,3-hexafluoropropoxy)-2,3,3,3-tetrafluoropropionate” or “ammonium difluoro{[2,2,4,5-tetrafluoro-5-(trifluoromethoxy)-1,3-dioxolan-4-yl]oxy}acetate”).tw,kf.	306
8	(PFESAs or “6:2 Cl-PFESA; F-53B” or “8:2 Cl-PFESA; PFESA-BP2, Nafion Byproduct 2” or ((Perfluoroalkyl or “6:2 chlorinated polyfluorinated” or “8:2 chlorinated polyfluorinated”) adj “ether sulfonic acid?”)).tw,kf.	47
9	(PASFs or POSF or (8:2 adj (FTOH or FTSA or monoPAP or diPAP)) or “6:2 FTSA” or PFOSA or FOSA or EtFOSA or FC-807 or (n:2 adj (FTOHs or FTSAs)) or FASAs or MeFASAs or EtFASAs or ((“perfluoroalkane sulfonyl” or perfluorooctanesulfonyl) adj fluoride?) or ((n:2 or 8:2 or 6:2) adj fluorotelomer adj (alcohol? or sulfonic acid? or phosphate monoester? or phosphate diester?)) or “n:2 Polyfluoroalkyl phosphoric acid esters” or (((perfluoroalkane or perfluorooctane) adj (sulfonamide? or sulfonamidoacetic acid?)) or perfluoroalkyl phosphate or “Ammonium bis[2-[N-ethyl (heptadecafluorooctane)” or “sulfonylamino]ethyl]phosphate”)).tw,kf.	852
10	(PASFs or POSF or (8:2 adj (FTOH or FTSA or monoPAP or diPAP)) or “6:2 FTSA” or PFOSA or FOSA or EtFOSA or FC-807 or (n:2 adj (FTOHs or FTSAs)) or PAPs or FASAs or MeFASAs or EtFASAs or ((“perfluoroalkane sulfonyl” or perfluorooctanesulfonyl) adj fluoride?) or ((n:2 or 8:2 or 6:2) adj fluorotelomer adj (alcohol? or sulfonic acid? or phosphate monoester? or phosphate diester?)) or “n:2 Polyfluoroalkyl phosphoric acid esters” or (((perfluoroalkane or perfluorooctane) adj (sulfonamide? or sulfonamidoacetic acid?)) or perfluoroalkyl phosphate or “Ammonium bis[2-[N-ethyl (heptadecafluorooctane)” or “sulfonylamino]ethyl]phosphate”)).tw,kf.	2751
11	((PFE adj (akane? or alkene?)) or Hostinert 216 or PMVE or PPVE or PEVE or Mv31 or Move3 or FC-3284 or PF-310 or Tetraconazole or Noviflumuron or Novec 7700 or (perfluoroether adj (alkane? or alkene?)) or “perfluoro(5,8,9,12-tetramethyl-4,7,10,13-tetraoxahexadecane)” or “perfluoro(5,6,9,12-tetramethyl-4,7,10,13-tetraoxahexadecane)” or “1,1,2-Trifluoro-2-(trifluoromethoxy)ethene” or “1,1,1,2,2,3,3-heptafluoro-3-[(trifluorovinyl)oxy]propane perfluoropropylvinylether” or “1,1,2-Trifluoro-2-(pentafluoroethoxy)ethene” or “1,1,2,2,3,3-hexafluoro-1-trifluoromethoxy-3-trifluorovinyloxypropane” or “1-[difluoro(trifluoromethoxy)methoxy]-1,2,2-trifluoroethylene” or “2,2,3,3,5,5,6,6-octafluoro-4-(trifluoromethyl)morpholine” or “1-[3-[4-((heptadecafluorononyl)oxy)-benzamido]propyl]-N,N,N-trimethylammonium iodide” or “1-[2-(2,4-Dichlorophenyl)-3-(1,1,2,2-tetrafluoroethoxy)propyl]-1H-1,2,4-triazole” or “2-(trifluoromethoxy)-benzenesulphonamide” or “2,2,3,3,5,5,6,6-octafluoro-4-(1,1,1,2,3,3,3-heptafluoropropan-2-yl)morpholine” or “2,2,3,3,5,5,6,6-octafluoro-4-(heptafluoropropyl)morpholine” or “(1 s,4r)-4-Propyl-4′-[4-(trifluoromethoxy)phenyl]-1,1′-bi(cyclohexyl)” or “2′,3,5-Trifluoro-4”-(trans-4-propylcyclohexyl)-4-trifluoromethoxy-[1,1′;4′,1″]terphenyl” or “2,3,3,4,4-pentafluoro-2,5-bis(1,1,1,2,3,3,3-heptafluoropropan-2-yl)-5-methoxytetrahydrofuran”).tw,kf.	170
12	(perfluorocarbon? or “F-gas*” or HFC-23 or HFC-125 or HFC-134a or Norflurane or HFC-143a or HFC-227ea or HFC-236fa or HFC-236ea or HFC-245fa or HFC-365mfc or HFC-4310mee or Vertrel XF or HFC-5213 or HFC-7613 or C6-ethane or HFCPA or ZEORORA or HFE-7100 or HFE-7200 or HFE-7300 or HFE-7500 or HFE-365pcf3 or HFO-1234yf or HFO-1243zf or “HFO-1234ze(E)” or “HFO-1336mzz(Z)” or “HFO-1336mzz(E)” or HFO-1114 or HFO-1216 or HFO-1132a or Vinylidenfluoride or hydrofluorocarbon? or “fluoroform, trifluoromethane” or pentafluoroethane or “1,1,1,2-Tetrafluoroethane” or “1,1,1-trifluoroethane” or “1,1,1,2,3,3,3-heptafluoropropane” or “1,1,1,3,3,3-hexafluoropropane” or “1,1,1,2,3,3-hexafluoropropane” or “1,1,1,3,3-pentafluoropropane” or “1,1,1,3,3-pentafluorobutane” or “1,1,1,2,2,3,3,4,4,5,5,6,6-tridecafluorohexane” or “1,1,1,2,2,3,3,4,4,5,5,6,6-tridecafluorooctane” or “1,1,2,2,3,3,4-heptafluorocyclopentane” or hydrofluoroether? or “1,1,1,2,2,3,3,4,4-Nonafluoro-4-methoxybutane - 2-[difluoro(methoxy)methyl]-1,1,1,2,3,3,3-heptafluoropropane (1:1)” or “1,1,1,2,2,3,4,5,5,5-Decafluoro-3-methoxy-4-(trifluoromethyl)pentane” or “3-Ethoxyperfluoro(2-methylhexane)” or “3-(Difluoromethoxy)-1,1,2,2-tetrafluoropropanec” or “hydrofluoroolefin?” or “2,3,3,3-Tetrafluoro-1-propene” or “3,3,3-trifluoropropene” or “(1E)-1,3,3,3-Tetrafluoro-1-propene” or “(2Z)-1,1,1,4,4,4-Hexafluoro-2-butene” or “(2E)-1,1,1,4,4,4-Hexafluoro-2-butene” or tetrafluoroethylene or hexafluoropropylene or “1-1-difluoroethene”).tw,kf.	4504
13	“2,2,2-Trifluoroethanol”.tw,kf.	676
14	(HFO or HFE or PFE or PAPs or TFE og HFC).tw,kf.	7299
15	or/3-14	20,190
16	2 or 15	20,191
17	epidemiologic studies/ or exp. case-control studies/ or exp. cohort studies/ or controlled before-after studies/ or cross-sectional studies/ or Controlled Before-After Studies/	2,739,581
18	(“non randomi*” or nonrandomi* or “case control” or casecontrol or Longitudinal or Retrospective or “panel study” or “panel studies” or crosssectional or crossectional or “cross sectional” or cohort analy* or “control group” or “case stud*”).tw,kf.	1,899,515
19	((cohort or controlled or comparative clinical) adj (study or studies or trial?)).tw,kf.	570,599
20	((observational or Follow up or epidemiologic$ or prospective) adj3 (study or studies or trial?)).tw,kf.	718,496
21	((“controlled before” adj2 after) or pretest or posttest or pre test or post test).tw,kf.	33,168
22	or/17-21	4,153,271
23	16 and 22	2976
24	(fluor* or perfluor* or polyfluor*).tw,kf.	818,697
25	23 and 24	1228
26	23 not 25	1748

Please see also List of Abbreviations, for description of Table see Methods section

**Table 3:** Functional immunotoxicity studies (T-cell Dependent Antibody Response (TDAR), T-cell Independent Antibody Response (TIAR) studies, host infection studies and lymphoproliferative response) with PFASs in rodents (see also Results, Repeated dose toxicity and immunotoxicity studies in animals section)

PFASs	Species, strain, sex, and group size	Dose, exposure duration, and exposure route	Experimental design and study protocol	Response and effects observed	Reference
PFOA	Mouse (C57BL/6, 4-6 M per group)	0 and 24 mg/kg bw/day for 10 days (diet)	**TDAR study**. Immunization to HRBC (i.v. injection with 200 μL of 5-10 × 10^7^ HRBC/mL in EBSS) on day 5, 6 days before sacrifice. One group continued PFOA treatment for 6 days after immunization (i.e. total of 16 days exposure; others normal chow). Measurement of HRBC-specific IgM levels with PFC assay and ELISA.	**TDAR study**. ↓ plaque formation by HRBC-specific IgM and IgG, ↓ HRBC-specific IgM and IgGs in serum, both at 24 mg/kg bw/day.	[69]
PFOS, PFOA	Mouse (Balb/c, 5 F per group)	0 and 20 mg/kg bw/day for 21 days (oral gavage)	**TDAR study**. Immunization to OVA twice (2 weeks apart, i.p. injections of 0.1 mg/kg with 100 μL OVA) on days 8 and 15. Second injection was 7 days before sacrifice. Measurement of OVA-specific IgM levels with ELISA. **Other observations:** Splenic and thymic cellularity.	**TDAR study.** ↓ OVA-specific IgM at 20 mg/kg bw/day PFOA and 20 mg/kg bw/day PFOS respectively.	[64]
PFOA	Mouse (Crl:CD-1(ICR)BR, 20 M per group) Rats, (Crl:CD(SD)IGS BR, 10 M per group)	0.3, 1, 10, and 30 mg/kg bw/day for 29 days (oral gavage, mouse and rat)	**TDAR study**. Immunization to SRBC (i.v. injection with 0.5 mL of 4 × 10^8^ SRBC/mL (rat) or 0.2 ml of 1 × 10^9^ SRBC/mL (mouse)) on day 23 (rat) or 24 (mouse), seven (rats) or six (mouse) days before sacrifice. Measurement of SRBC-specific IgM levels with ELISA. **Other observations.** Body weight, organ weight and histopathology, hematology, clinical chemistry, corticosterone measurement, spleen and thymus cellularity.	Mouse: **TDAR study.** ↓ SRBC-specific IgM ≥ 10 mg/kg bw/day. **Other observations.** ↑ liver weight and liver focal necrosis ≥1 mg/kg bw/day. ↓ body weight, spleen and thymus weight, atrophy of lymphoid tissue, eosinophils, total number of thymocytes and splenocytes ↑ neutrophils and monocytes, corticosterone serum levels) ≥ 10 mg/kg bw/day. ↓ lymphocytes at 30 mg/kg bw/day. Rat: **TDAR study.** PFOA had no effect on production of anti-SRBC IgM. **Other observations**. ↑ liver weight, minimal focal liver necrosis and corticosterone serum levels ≥10 mg/kg bw/day.	[65]
	PFOA	Mouse (C57BL/6 N, 8 F per group)	0 and 30 mg/kg for 15 days (constant group) or 10 days (recovery group); dose response study I with 0, 3.75, 7.5, 17, 30 mg/kg bw/day for 15 days (drinking water); and dose response study II 0, 0.94, 1.88, 3.75, 7.5 mg/kg bw/day for 15 days (drinking water)	**TDAR study**. Immunization to SRBC once (i.v. injection with 4.0 × 10^7^ SRBC in 0.2 mL saline), on day 11, or immunization to SRBC twice (2 weeks apart, i.v. injection with 4.0 × 10^7^ SRBC in 0.2 mL saline), on day 11 and day 25. Animals were sacrificed 5 days after. Measurement of SRBC-specific IgM and IgG levels with ELISA. **DTH study.** On day 11, animals were sensitized with a subcutaneous injection of BSA-CFA (0.05 mL of 2 mg/mL BSA-CFA). At day 18, animals were challenged with an injection of 0.05 mL of heat-aggregated BSA into the right footpad. Footpad thickness was measured 24 h post-challenge. **Other observations.** Body weight, lymphoid organ weights (spleen, thymus).	[66]
PFOA	Mouse (adrenalectomized or sham-operated C57BL/6 N, 6 F per group)	0, 3.75, 7.5, or 15 mg /kg bw for 10 days (drinking water)	**TDAR study.** Immunization to SRBC (i.v. injection with 7.5 × 10^7^ SRBC in 0.2 mL saline) on day 11, 5 days before sacrifice. Measurement of SRBC-specific IgM levels with ELISA. **Other observations.** Corticosterone serum levels.	**TDAR study.** ↓ IgM ≥ 7.5 mg/kg bw/day in adx and ↓ IgM at 15 mg/kg bw/day in sham mice. **Other observations.** Increase in corticosterone only at 15 mg/kg bw/day in sham mice. Hence, suppression of SRBC-specific IgM was not the result of corticosterone production.	[67]
PFOA	Mouse (PPARα-KO; B6.129S4-Ppartm1GonzN12 and WT C57BL/6-Tac, 4-6 F per group) TIDAR: mouse (C57BL/6 N WT, 8 F per group)	PPARα-KO mice compared to WT, exposed to 0, 7.5 or 30 mg/kg bw/day for 15 days (TDAR) or 0, 0.94, 1.88, 3.75, and 7.5 mg/kg bw/day for 15 days (TIDAR) (drinking water)	**TDAR study.** Immunization to SRBC (i.v. injection with 7.5 × 10^7^ SRBC in 0.2 mL saline) on day 11, 5 days before sacrifice. Measurement of SRBC-specific IgM levels with ELISA. **TIDAR study.** Immunization to DNP-LPS (i.v. injection with 1 μg DNP-LPS in 0.2 mL saline) on day 11, seven days before sacrifice. Measurement of DNP-LPS-specific IgM levels with ELISA. **Other observations.** Body weights, lymphoid organ weights, splenic lymphocyte phenotypes (the latter in non-immunized PFOA-treated mice).	**TDAR study.** ↓ SRBC-specific IgM at 30 mg/kg bw/day in both PPARα KO (no decline in bw, spleen-, or thymus weight observed) and WT mice (decline in bw, spleen- and thymus weight). **TIDAR study.** ↓ DNP-LPS-specific IgM ≥ 1.88 mg/kg bw/day.	[116]
PFOA	Mouse (B6C3F1, 12-16 F per group)	0, 1.88 and 7.5 mg/kg bw/day for 28 days (drinking water)	**TDAR study.** Immunization to KLH (i.p. injection with 300 mg KLH/mice in a total volume of 0.5 mL) on day 24, 5 days before sacrifice. Measurement of KLH-specific IgM levels with ELISA. **Other observations.** Body weight, serum cytokines, serum corticosterone.	**TDAR study.** ↓ KLH-specific IgM ≥ 1.88 mg/kg bw/day. **Other observations.** Serum corticosterone was not significantly correlated with TDAR or measured cytokines. At 5 mg/kg bw/day, ↓ Th2, mixed response for Th1 cytokines (overall favoring a Th1 balance). At both treated groups, ↓ pro-inflammatory cytokines.	[63]
PFOS	Mouse (B6C3F1, 5 per sex/group for TDAR study, 10 F per group for TIDAR study)	0, 0.166, 1.66, 3.31, 16.6, 33.1, and 166 μg/kg bw/day for 28 days (oral gavage) (TDAR study) 0 and 334 μg/kg bw/day for 21 days (oral) (TIDAR study)	**TDAR study.** Immunization to SRBC (i.p. injection, 0.1 mL of 25% SRBC in PBS) on day 23, 5 days before sacrifice. Measurement of SRBC-specific IgM with PFC assay. **TIDAR study.** Immunization to TNP-LPS (i.v. injection in tail vein, 100 μL of 1 μg TNP-LPS/μL) on day 14, 7 days before sacrifice. Measurement of TNP-LPS-specific IgM with ELISA. **Other observations.** Splenic and thymic CD4/CD8 subpopulations.	**TDAR study.** ↓ SRBC-specific IgM ≥ 1.66 μg/kg bw/day in M and ≥ 16.6 μg/kg bw/day in F. **TIDAR study.** ↓ TNP-specific IgM at 334 μg/kg bw/day in F. **Other observations.** Alteration of splenic (but not thymic) CD4/CD8 T-cells ≥3.31 μg/kg bw/day in M, and alteration of splenic and thymic CD4/CD8 T-cells ≥3.31 μg/kg bw/day in F.	[70]
PFOS	Mouse (C57BL/6, 12 M per group)	0, 5, 20 and 40 mg/kg bw/day for 7 days (oral gavage)	**TDAR study.** Immunization to SRBC (i.p. injection with 0.1 mL of a 25% SRBC suspension in PBS) on day three, 5 days before sacrifice. Measurement of SRBC-specific IgM with PFC assay. **NK cell activity.** Splenocytes and Yac-1 cells were prepared in the ratio 10:1. Then, the amount of LDH released from lysed Yac-1 cells was determined to observe NK cell activity. **Lymphoproliferative response.** Isolated splenocytes were exposed to either 10 μg/mL ConA or LPS. After that, proliferation was determined using the MTT assay. **Other observations.** Body weight, organ weights (liver, kidney, spleen, and thymus), thymus and spleen cellularity, serum corticosterone, thymic and splenic CD4/CD8 subpopulations.	**TDAR study**. ↓ SRBC-specific IgM ≥ 5 mg/kg bw/day. **NK cell activity.** ↓ NK cell activity ≥20 mg/kg bw/day **Lymphoproliferative response.** ↓ splenic leukocyte proliferation in response to ConA and LPS ≥ 5 mg/kg bw/day. **Other observations**. ↓ splenic and thymic cellularity, ↑ liver weight ≥ 5 mg/kg bw/day. ↓ body weight, thymus and spleen weight, ↓ splenic and thymic lymphocyte subpopulations, ↑ serum corticosterone ≥20 mg/kg bw/day. Severe impairment of terminal body weight and food intake ≥20 mg/kg bw/day. Mean terminal body weight decreased with approximately 15 and 25% at 20 and 40 mg/kg bw/day respectively.	[71]
PFOS	Mouse (C57BL/6, 10 M per group)	0, 0.008, 0.08, 0.42, 0.83, and 2.1 mg/kg bw/day for 60 days (oral gavage)	**TDAR study.** Immunization to SRBC (i.p. injection with 0.1 mL of a 25% SRBC suspension in PBS) on day 57, 4 days before sacrifice. Measurement of SRBC-specific IgM with PFC assay. **NK cell activity.** Splenocytes and Yac-1 cells were prepared in the ratio 10:1. Then, the amount of LDH released from lysed Yac-1 cells was determined to observe NK cell activity. **Lymphoproliferative response.** Isolated splenocytes were exposed to either 10 μg/mL ConA or LPS. After that, proliferation was determined using the MTT assay. **Other observations.** Body weight, organ weights (liver, spleen, and thymus), thymus and spleen cellularity, serum corticosterone, thymic and splenic CD4/CD8 subpopulations.	**TDAR study.** ↓ SRBC-specific IgM ≥ 0.08 mg/kg bw/day. **NK cell activity**. ↓ NK cell activity ≥0.83 mg/kg bw/day. **Lymphoproliferative response.** ↓ splenic leukocyte proliferation in response to ConA and LPS ≥ 0.83 mg/kg bw/day. **Other observations.** ↓ body weight, spleen and thymus weights, spleen and thymus cellularity, splenic and thymic T-cell subpopulations ≥0.42 mg/kg bw/day. ↑ serum corticosterone level ≥ 0.83 mg/kg bw/day.	[72]
PFOS	Mouse (C57BL/6, 6 M per group)	0, 0.0083, 0.017, 0.083, 0.42 and 0.83 mg/kg bw/day for 60 days (oral gavage)	**TDAR and DTH study.** Immunization to SRBC (i.v. injection with 4.0 × 10^7^ SRBC in 0.2 mL saline) on day 54 in 12 animals per group, 7 days before sacrifice. On day 60, 6 animals per group received a SRBC booster immunization (footpad injection with 4.0 × 10^7^ SRBC in 0.2 mL saline) for assessment of a DTH response (footpad swelling) and different immunoglobulin determinations (IgGs, IgE). Measurement of SRBC-specific immunoglobulins with ELISA. **Other observations.** Body weight, organ weights (liver, spleen, and thymus), serum corticosterone, measurement of cytokines.	**TDAR study.** ↓ SRBC-specific IgM ≥ 0.083 mg/kg bw/day. **DTH study.** No increased footpad swelling was observed in treated groups compared to the control group. **Other observations.** ↓ bw change, food consumption, spleen and thymus weights ≥0.833 mg/kg bw/day. ↑ SRBC-specific IgGs and SRBC-specific IgE at 0.83 mg/kg bw/day. ↑ IL-4 ≥ 0.083 mg/kg bw/day, ↑ IL-10 at 0.83 mg/kg bw/day, ↓ IL-2 and INF-γ at 0.83 mg/kg bw/day.	[73]
PFOS	Mouse (B6C3F1, 10-12 dams per group)	0.1, 1, and 5 mg/kg bw/day on GD 1-17 (oral gavage)	**TDAR study.** Immunization to SRBC (i.v. injection with 7.5 × 10^7^ SRBC in 0.2 mL saline) in eight-week-old F1 pups (6 sex/dose), 4 days before sacrifice. Measurement of SRBC-specific IgM with PFC assay. **NK cell activity.** Splenocytes and Yac-1 cells were prepared in different ratios (200:1, 100:1, 50:1, 25:1, 12.5:1, 6.25:1). Then, lysis was determined by lysing ^51^Cr-labeled Yac-1 cells with 0.1% Triton X in complete media. **Other observations.** Body weight, organ weight (liver, thymus, spleen, uterus), spleen and thymus cellularity, splenic and thymic CD4/CD8 subpopulations, nitrite production by peritoneal macrophages.	**TDAR study.** ↓ of SRBC-specific IgM in M at 5 mg/kg bw/day at week 8. **NK cell activity.** ↓ NK cell activity in M ≥ 1 mg/kg bw/day and in F at 5 mg/kg bw/day at week 8. **Other observations.** At week 4, ↑ liver weight in M at 5 mg/kg bw/day. ↓ CD3^+^ and CD4^+^ thymocytes in M at 5 mg/kg bw/day. In maternal animals ↓ CD4:CD8 ratio at 5 mg/kg bw/day.	[74]
PFOS	Rat (SD, 10-15 per sex/group)	0.14, 1.33, 3.21, 6.34 (M) and 0.15, 1.43, 3.73, 7.58 (F) mg/kg bw/day for 28 days (diet)	**TDAR and DTH study.** Immunization to KLH twice (i.p. injection with 1 mg KLH) on day 14 and day 21, 14 and 7 days before sacrifice. On day 28, rats were injected with 2 mg heat-inactivated KLH or saline in the left and right footpad respectively, to measure swelling after 24 h. Right after measuring footpad swelling, blood serum was sampled for measuring KLH-specific IgG with ELISA. **Other observations.** Body weight, organ weights (liver, thymus, spleen), organ histopathology (liver, thymus, spleen, mesenteric lymph nodes), total serum immunoglobulin (IgM, IgA, IgG, unchallenged), peripheral blood lymphocyte phenotyping, splenocyte proliferation.	**TDAR study.** ↑ KLH-specific IgG in M at the highest dose in M, but not in F. **DTH study.** There was no statistically significant change in footpad swelling. **Other observations**. ↓ body weight and ↑ liver weight at the two highest dose groups (M, F), and ↓ spleen (M) and thymus (M, F) weight at the highest dose. ↑ total IgM (unchallenged) in F at the highest dose. No effect on peripheral blood lymphocyte phenotype, or splenic leukocyte proliferation in response to ConA and LPS. Challenging regimen and timepoint of measuring KLH-specific IgG differs from other TDAR studies.	[75]
PFOS	Mouse (B6C3F1, 5 M per group)	0 and 250 μg/kg bw/day for 28 days (diet)	**TDAR study.** Immunization to SRBC (i.p. injection with 0.1 mL of a 1:10 SRBC suspension in PBS) on day 23, 5 days before sacrifice. Measurement of SRBC-specific IgM and IgG with ELISA and IgM with PFC assay. **TIDAR study.** Immunization TNP-LPS (i.v. injection with 0.1 mL of 100 μl/mL TNP-LPS in 0.9% NaCl) on day 23, 5 days before sacrifice. Measurement of TNP-LPS-specific IgM with ELISA. **Other observations.** Organ and tissue weights (liver, epididymal fat, spleen, and thymus), serum corticosterone, immunophenotyping of the thymus and the spleen.	**TDAR, TIDAR, and other observations.** No change in the TDAR or TIDAR at 250 μg/kg bw/day compared to the control group. Serum levels of SRBC-specific IgM and IgG or levels of TNP-LPS-specific IgM were not altered by PFOS treatment. ↑ weight of the liver at 250 μg/kg bw/day. Cellular compositions of the thymus and spleen were not altered. No effect on corticosterone levels observed compared to the control group. **Study was performed with one low dose and had small group sizes.**	[78]
HFPO-DA	Mouse (C57BL/6, 6 per sex/group)	0, 1, 10, or 100 mg/kg bw/day for 28 days (oral gavage)	**TDAR study.** Immunization to SRBC (i.v. injection with 4 × 10^7^ SRBC in 0.2 mL saline) on day 24, 5 days before sacrifice. Measurement of IgM by ELISA. **Other observations.** Body weight, organ weights (liver, thymus, spleen), immunophenotyping of spleen.	**TDAR study.** ↓ SRBC-specific IgM in F, but not in M, at 100 mg/kg bw/day. **Other observations.** ↑ CD8^+^ and CD4^−^/CD8^−^ T cells in M at 100 mg/kg bw/day. ↓ relative spleen weight in F at 100 mg/kg bw/day.	[76]
PFMOAA	Mouse (C57BL/6, 4-6 per sex/group)	0, 0.00025, 0..025 and 2.5 mg/kg bw/day for 30 days (drinking water)	**TDAR study.** Immunization to SRBC (i.v. injection with 4 × 10^7^ SRBC in 0.2 mL saline) on day 26, 5 days before sacrifice. Measurement of IgM with ELISA. **Other observations.** Body weight, organ weights (liver, thymus, spleen), immunophenotyping of thymus and spleen.	**TDAR study.** No statistical differences were detected in the TDAR in M or F animals given PFMOAA compared to the control group. **Other observations**. No statistically significant effect was observed on body weight, lymphoid organ weight, and thymus and spleen immunophenotyping, up to the highest dose tested.	[79]
PFMOPrA	Mouse (C57BL/6, 4-6 per sex/group)	0, 0.5, 5, and 50 mg/kg bw/day for 30 days (drinking water)	**TDAR study.** Immunization to SRBC (i.v. injection with 4 × 10^7^ SRBC in 0.2 mL saline) on day 26, 5 days before sacrifice. Measurement of IgM with ELISA. **NK cell activity.** YAK-1 cells were prepared 5 days before the NK cell assay. Spleens were processed and lymphocytes were isolated. Lymphocyte and YAK-1 cells were prepared in three ratios (5:1, 10:1, and 30:1). After that, the percent specific lysis was determined by flow analysis. **Other observations.** Body weight, organ weights (liver, thymus, spleen), immunophenotyping of thymus and spleen.	**TDAR study.** No statistical differences were detected in the TDAR in M or F animals given PFMOPrA compared to the control groups. **NK cell activity.** There was no statistically significant change in NK cell activity. **Other observations.** ↓ in spleen weight in F at 0.5 and 50 mg/kg bw/day, ↑ in spleen weight in F at 5 mg/kg bw/day. **Dosing regimen of M animals was increased (2-fold) after week one of the experiment.**	
PFMOBA	Mouse (C57BL/6, 4-6 per sex/group)	0, 0.5, 5, and 50 mg/kg bw/day for 30 days (drinking water)	**TDAR study.** Immunization to SRBC (i.v. injection with 4 × 10^7^ SRBC in 0.2 mL saline) on day 26, 5 days before sacrifice. Measurement of IgM with ELISA. **NK cell activity.** YAK-1 cells were prepared 5 days before the NK cell assay. Spleens were processed and lymphocytes were isolated. Target cells (500 μL) were added to effector cells in three E:T ratios (5:1, 10:1, and 30:1). After that, the percent specific lysis was determined by flow analysis. **Other observations.** Body weight, organ weights (liver, thymus, spleen), immunophenotyping of thymus and spleen, natural killer cell activity.	**TDAR study.** No statistical differences were detected in the TDAR in M or F animals given PFMOBA compared to the control group. **NK cell activity**. There was no statistically significant change in NK cell activity. **Other observations**. ↑ B cells and NK cells in the spleen in M at all doses compared to the control group, and ↓ B cells and NK cells in the spleen at 50 mg/kg bw/day in F compared to the control group.	
PFOA	Mouse (C57BL/6, 4-6 per sex/group)	0, and 7.5 mg/kg bw/day for 30 days (drinking water)	**TDAR study.** Immunization to SRBC (i.v. injection with 4 × 10^7^ SRBC in 0.2 mL saline) on day 26, 5 days before sacrifice. Measurement of IgM with ELISA. **Other observations.** Body weight, organ weights (liver, thymus, spleen), immunophenotyping of thymus and spleen.	**TDAR study.** No statistical differences were detected in the TDAR in M or F animals given PFOA compared to the control group. **Other observations**. ↓ relative spleen weight at 7.5 mg/kg bw/day in F. **PFOA, serving as the positive control, was also negative in the TDAR study.**	
AFFF formulation (containing C5-C10 PFSA, PFOA, Cl-PFOS or precursors thereof)	Mouse (C57BL/6, 6 per sex/group)	AFFF formulation (based on 0, 1.88, 3.75, 7.5, or 10 mg/kg bw/day PFOS + PFOA measured in the formulation) for 10 days followed by 6 days of depuration (oral gavage)	**TDAR study.** Immunization to SRBC (i.v. injection with 7.5 × 10^7^ SRBC in 0.2 mL saline) on day 11, 5 days before sacrifice. Measurement of SRBC-specific IgM levels with ELISA. **Other observations.** Body weight, liver weight, lymphoid organ weights, spleen cellularity, splenic lymphocyte subpopulations.	**TDAR study.** ↓ SRBC-specific IgM in F and M ≥ 7.5 mg/kg bw/day. **Other observations.** ↓ rel. Spleen weight in M at 10 mg/kg bw/day and ↓ body weight, rel. Thymus weight in F and M ≥ 7.5 mg/kg bw/day. ↑ liver weight in all treated dose groups in M and F compared to the control groups.	[77]
PFOA	Mouse (C57BL/6, 6 per sex/group)	0 and 7.5 mg/kg bw/day PFOA) for 10 days followed by 6 days of depuration (oral gavage)	**TDAR study.** Immunization to SRBC (i.v. injection with 7.5 × 10^7^ SRBC in 0.2 mL saline) on day 11, 5 days before sacrifice. Measurement of SRBC-specific IgM levels with ELISA.	**TDAR study.** ↓ SRBC-specific IgM in F and M at 7.5 mg/kg bw/day.	
PFDA	Mouse (B6C3F1/N, 8 F) and rat (SD, 8 F)	Rats: 0, 0.125, 0.25, 0.5, 1, and 2 mg/kg bw/day for 28 days (oral gavage) Mice: once each week (days 1, 8, 15, and 22) at doses of 0, 0.3125, 0.625, 1.25, 2.5, and 5 mg/kg bw/week (oral gavage)	**TDAR study.** Immunization to SRBC and KLH (i.v. injection with 2 mg KLH and an unknown quantity of SRBC respectively) on day 23 (in rat and mouse), and measurement of anti-SRBC and anti-KLH with ELISA. In a separate assay, immunization to SRBC on day 25 and measurement of spleen IgM with APC response. **NK cell activity.** Splenocytes and ^51^Cr-labelled YAC-1 cells were prepared in different ratios (200:1, 100:1, 50:1, 25:1, 12.5:1, 6.25:1). Then, 100 μL supernatant was counted using a γ-counter. **DTH study.** On days 21 and 29, animals were challenged with a subcutaneous injection of *C. albicans* ((2 × 10^7^ organisms for rats, 1 × 10^7^ for mice) in the right footpad. Footpad thickness was measured right before the second challenge, and 24 h post-challenge. **Mononuclear phagocyte system (MPS) activity. Uptake and vascular clearance of**^51^Cr-labelled SRBC by fixed macrophages in the liver, spleen, thymus, lung and kidney. Intravenous injection with ^51^Cr-labelled SRBC on day 29. SRBC serum half-life and relative organ uptake was determined using a γ-counter 30-60 min. Post-injection. **Host resistance to infection study.** Mice (treated for 28 days as described above) were infected intranasally with *Influenza A/*Hong Kong*/8/86* (H3N2) virus at three challenge levels (1:2420, 1:440, and 1:80 dilutions). Mice were observed twice daily for 21d for changes in appearance, locomotion, and respiration. **Other observations.** Body weight, organ histopathology, total and differential white blood cell counts, spleen cell immunophenotyping, bone marrow DNA synthesis, colony formation, and differentials.	**TDAR study (mouse and rat).** No PFDA exposure-related effects were observed on the AFC response to SRBC in rats or mice, or in the serum IgM levels to SRBC or KLH in rats. **Host resistance to infection (mouse).** Treatment with PFDA did not decrease survival compared to the control during the 21 day post-challenge observation period. **DTH (mouse and rat).** No increased footpad swelling was observed in treated groups compared to the control group. **MPS activity (rat).** Altered functional activity of mononuclear phagocytic system in liver and thymus ≥0.25 mg/kg bw/day. **Other observations (mouse).** ↓ spleen weight, splenic atrophy (20%), ↓ total spleen cells, Ig + and NK+ cells at 5.0 mg/kg bw/week PFDA, and ↓ CD3+, CD4+, CD8+, and Mac3+ cells in the spleen ≥1.25 mg/kg bw/week PFDA. **Other observations (rat).** No change from controls on lymphoid organ weights, leukocyte subpopulations, and bone marrow cellularity.	[82]
PFHxS	Rat (HanTac: WH, dams, 8 or 20 litters per group in two separate experiments)	Dams were exposed to 0, 25, or 45 mg/kg bw/day a-nd 0, 0.5, 5 and 25 mg/kg bw/day PFHxS in two separate experiments during GD7-PND22 (oral gavage)	**TDAR study.** Immunization to KLH in weaned offspring twice (14 days apart, 200 μL of 1.5 mg/ml KLH via i.p. injection). At PND28 and PND 37 in experiment 1 and at PND34 and PND43 in experiment 2. Measurement of KLH-specific IgM and IgG with ELISA. **DTH study.** The day before sacrifice, animals were challenged intradermally with an injection of 20 μL of 5 mg/ml KLH or 20 μL saline in the left and right ear respectively. Right after sacrifice, the thickness of the ear was measured and weighted. **Other observations.** Lymphoid organ weights.	**TDAR study.** No effects on IgM and IgG responses up to the highest dose tested compared to the control group. **DHT study.** No increased ear swelling was observed in treated groups compared to the control group. **Other observations.** No effects on lymphoid organs in M or F at any dose apart from ↓ in lymph node wt in M at PND 16 in 25 and 45 mg/kg bw/day dose groups in experiment 1. Challenging regimen and timepoint of measuring KLH-specific IgM and IgG differs from other TDAR studies. Also the positive control cyclophosphamide was negative.	[80]
PFOS	Mouse (C57BL/6, sex not specified, 4 per group)	0 and 2 mg/kg bw/day for 18 days (oral gavage)	**Host resistance to infection study**. Challenge with mouse *Citrobacter rodentium* bacterium strain DBS100 (10^10^ cfu) in 200 μL PBS at day 7 via oral gavage. PFOS exposure was continued until the end of the experiment. Intestinal lamina propria lymphocytes were harvested during exposure and infection.	**Host resistance to infection study**. At 2 mg/kg bw/day, PFOS inhibited the outgrowth of the pathogen (↑ of IL-22 from ILC3 cells). In the later phase ↑ bacterial counts and ↑ of inflammatory cytokines, (↑ of IL-22 and IL-17 from ILC3 cells, ↑ IFN-γ from CD3^−^ cells, ↑ of IL-22 and IL-17 from Th17 cells), reduced mucus production, dysbiosis, ↑ levels of *E.coli*.	[83]
PFOS	Mouse (C57BL/6, 4-5 per sex/group (exp. 1) and 8-10 per sex/group (exp. 2))	0 and 1.5 μg/kg bw/day for 28 days (oral gavage)	**Host resistance to infection study.** **Experiment 1:** Challenge with mouse influenza virus strain A/WSN/33 (H1N1) intranasally (1e^6^ pfu/mouse) at day 28. Body weights were monitored. After 11 days, BALF was collected from the lungs, and blood, lungs, liver and spleen were collected. **Experiment 2:** Challenge with mouse influenza virus strains A/WSn/33(H1N1)(WSN-OVA_1_) and A/WSN/33(H1N1)(WSN-OVA_11_) intranasally (2e^4^ pfu/mouse) at day 28. Body weights and tissue collections were as described above.	**Host resistance to infection study.** **Experiment 1.** No effect on virus-induced weight loss, on the number of inflammatory cells, T cells, and granulocytes in the lung. ↓ CD4^+^ and ↑ CD8^+^ T cells in BALF and ↑ CD4^+^CD44^Hi^ in the lung at 1.5 μg/kg bw/day. **Experiment 2.** No effect on virus-induced weight loss, no effect on inflammatory cells in BALF, no difference in the percentage of antigen-specific CD4^+^ or CD8^+^ T cells in the spleen. ↑ number of antigen-specific CD4^+^ T cells in the spleen at 1.5 μg/kg bw/day. **Study was performed with one low dose and had small group sizes.**	[81]
PFNA	Mouse (C57BL/6, 5 per sex/group).	0 or 0.46 mg/kg bw once (i.p. injection)	**Lymphoproliferative response.** Immunization to LPS on day 14 (i.p. injection with 1 mg/kg LPS or saline). 1.5 hours after administration of LPS, blood was collected to quantify the TNFα concentration by ELISA. **Other observations.** Spleen, thymus, kidney and liver were collected on day 14. Splenocyte and thymocyte immunophenotyping was performed by flow cytometry analysis.	**Lymphoproliferative response.** ↑ TNFα **Other observations.** Spleen atrophy, ↓ spleen weight, spleen leukocyte count, spleen red blood cell count, ↑ CD4^+^, CD8^+^ in the spleen, ↓ CD4^+^/CD8^+^ cells in the thymus, ↑ CD4^+^, CD8^+,^ CD4^−^/CD8^−^ cells in the thymus. ↓ CD14^+^ cells and CD19^+^ cells in the spleen. **Animals were exposed once via i.p. injection.**	[97]
PFNA	Mouse (C57BL/6 J, 5 per sex/group).	0 or 0.46 mg/kg bw once (i.p. injection)	**Lymphoproliferative response.** Immunization to LPS on day 28 (i.p. injection with 1 mg/kg LPS or saline). 1.5 hours after administration of LPS, blood was collected to quantify the TNFα concentration by ELISA. **Other observations:** Spleen, thymus and liver were collected on day 28. Splenocyte and thymocyte immunophenotyping was performed by flow cytometry analysis.	**Lymphoproliferative response**. ↑ TNFα **Other observations.** ↑ CD14^+^ cells and ↓ CD19^+^ cells in the spleen. Spleen and thymus atrophy, ↓ spleen and thymus weights, ↑ CD4^+^, CD8^+^ in the spleen, ↓ CD4^+^/CD8^+^ cells in the thymus**.** Animals were exposed once via i.p. injection.	[96]

Results are described in Results, Repeated dose toxicity and immunotoxicity studies in animals section

↓ reduction (suppression), ↑ increase, *AFC* Antibody forming cell, *BALF* Bronchoalveolar lavage fluid, *BSA-CFA* Bovine serum albumin in complete Freund’s adjuvant, *CD* Cluster of differentiation, *ConA* Concanavalin A, *DNP* 2,4-dinitrophenyl, *DTH* Delayed-type hypersensitivity, *EBSS* Earle’s balanced solution, *F* Female, *HFPO-DA* Hexafluoropropylene oxide-dimer acid, *HRBC* Horse red blood cells, *IgM* Immunoglobulin M, *i.p.* Intraperitoneal, *i.v.* Intravenous, *KLH* Keyhole limpet hemocyanin, *KO* Knock-out, *LDH* Lactate dehydrogenase, *LPS* Lipopolysaccharide, *M* Male, *NK* Natural killer, *OVA* Ovalbumin, *PBS* Phosphate-buffered saline, *PFBA* Perfluorobutanoic acid, *PFBS* Perfluorobutane sulfonic acid, *PFC* Plaque forming cell, *PFDA* Perfluorodecanoic acid, *PFHxA* Perfluorohexanoic acid, *PFHxS* Perfluorohexane sulfonic acid, *PFNA* Perfluorononanoic acid, *PFMOAA* Perfluoro-2-methoxyacetic acid, *PFMOPrA* Perfluoro-2-methoxypropanoic acid, *PFMOBA* Perfluoro-4-methoxybutanioc acid, *PFOA* Perfluorooctanoic acid, *PFOS* Perfluorooctane sulfonic acid, *PND* Postnatal day, *PPARα* Peroxisome proliferator activated receptor alpha, *SRBC* Sheep red blood cells, *TAD* Total administered dose, *TDAR* T-cell dependent antibody response, *TIAR* T-cell independent antigen response, *TNFα* Tumor necrosis factor alpha, *TNP* 2,4,6-trinitrophenyl., *WT* Wild-type

**Table 4:** Experimental studies observing modulation of NF-κB by PFAS (see also Modulation of NF-κB regulated gene transactivation section, Results)

PFAS	Study / Method	Effect	Reference
PFNA	**In vivo**: p.o. 14 day treatment of mice (2008) and rats (2009)	No effect on NF-κB observed measured with RT PCR of thymus cells;	[93, 94]
PFOS, PFOA	**In vitro**: THP-1 cells, treated with 10-100 μg/ml PFOA and 1-100 μg/ml PFOS in the presence of LPS for 30 min or 3 hrs (NF-κB promoter activity);	↓ of NF-κB by PFOS and PFOA in a dose dependent manner PFOS: ↓ of LPS-induced I-κB activation, NF-κB binding to DNA, p65 phosphorylation, and transcription according (independent of PPARα); PFOA: ↓ p65 phosphorylation and NF-κB mediated transcription (through PPARα)	[205, 206]
PFOS	**In vivo**: BALB/c mice, regular (RD) or high-fat diet (HFD); then exposed to PFOS (0, 5, and 20 mg/kg/day) for 14 days. (to investigate interference with lipid metabolism)	RD group: atrophy of immune organs, bw ↓ (highest dose), histopathological alterations of thymus and spleen, ↑ apoptosis of thymocytes HFD group: More serious atrophy was seen in the immune organs; PFOS exposure did not suppress the NF-κB signalling pathway (↑IL-1β)	[207]
PFBS, PFOS, PFOA, PFDA, fluorotelomer	**In vitro** promyelotic cell line THP-1 and PHA-stimulated human PBLs; Cells treated with 0.1 – 10 μg/ml PFAS and LPS for 30 min or 3 hrs (NF-κB promoter activity);	↓of LPS-induced phosphorylation of p65 (RELA) and NF-κB driven gene transcription (only PFOA activated PPARα) PFBS and PFDA prevented LPS-induced I-κB degradation All PFCs ↓ TNFα, while effects on other cytokines where unequal	[206]
PFOA	**In vitro**: HMC-1 cells, 50-400 μM PFOA for 12 hrs; proteins measured by Western blot. [in vivo mouse allergy model; 10 and 50 mg/kg bw PFOA for 4 days on dorsal surface of each ear]	In vitro: ↑ of pro-inflammatory cytokines (TNF-α, IL-1β, IL-6, and IL-8) was NF-κB dependent; ↑ of: phosphorylation of p38, translocation of p65 NF- κB to the nucleus and degradation of IκB) *[*In vivo: ↑IgE-dependent allergic local reaction in mice]	[128]
PFOA	**In vivo*****:*** zebrafish (0.05, 0.1, 0.5, and 1 mg/L) for 21 d, expression analysis of spleen	↑pro-inflammatory cytokine (IL-1β and IL-21) at a low exposure concentration (0.05 mg/L) and ↓at higher exposure concentrations (≥ 0.1 mg/L) via Myd88/NF-κB pathway ↓TLR2 expression (at 1 mg/L) to 56% compared to control	[112]
PFDA	**In vitro**: human AGS cell line treated with 5-50 μM PFDA for 24 hrs, protein and mRNA level	↑ NF-κB activity at 5 μM, also c-Rel and p52 were ↑; (↑IL-13, IL-18 and NLRP3)	[208]
PFDA (C = 10) and PFUnDA (C = 11), no effect: PFHpA C = 7, PFNA C = 9;	**In vitro**: IgE stimulated mast cells, RBL -2H3, transfected with NF-κB luciferase transporter construct Treated with respective PFAS 100 μM, 30 min [In vivo: ovalbumin induced system of anaphylaxis]	In vitro: IgE stimulated mast cells: ↑ pro-inflammatory cytokines (PFDA and PFUnDA only) ↑intracellular Ca, ↑ histamine RBL cells: ↑ NF-κB activity (PFDA and PFUnDA only) *[*In vivo: ↑allergic reactions only with PFDA, PFNA and PFUnDA]	[111]
PFOS	**In vitro**: IgE stimulated RBL-2H3 cells; treated with 100 and 500 μM PFOS for 30 min; assayed with Western blot and luciferase assay [In vivo: OVA induced anaphylaxis ICR mouse model (50-150 mg/kg PFOS p.o. 3x on day 9, 11 and 13)]	In vitro: ↑ NF-κB (luciferase activity); ↑ degradation of I-κB and nuclear translocation of NF-κB; ↑ gene expression of pro-inflammatory cytokines; pre-treatment with a NF-κB inhibitor: ↓TNF-α production and luciferase activity; *[*In vivo: allergic symptoms were ↑ by PFOS]	[160]
PFOS	**In vitro**: rat primary KCs and hepatocytes treated with 100 μM PFOS for 48 hrs **In vivo**: male SD rats 1 or 10 mg/kg bw PFOS per gavage for 20 days;	In vitro: KC cells - ↑ NF-κB activation and p65 translocation. ↑ I-κB and JNK phosphorylation in hepatocytes and KCs; ↑ production of TNF-α and IL-6 in KCs, and was ↓ by NF-kB inhibitor ↑ hepatocyte proliferation by altering regulatory proteins (↑ PCNA, c-Jun, c-MYC and CyD1 in vitro & in vivo) In vivo: hepatocellular damage and inflammation, ↑serum TNFα and IL-6 level	[209]
PFOA and PFOS	**In vitro**: macrophages treated with six EDCs via sirtuin (SIRT) regulation using the murine macrophage RAW 264.7 cell line	PFOS and PFOA did not alter NF-κB expression (only Mono(2-ethylhexyl) phthalate did)	[210]
PFOS	In vivo (hepatotoxicity and immunotoxicity) in zebrafish: 0, 0.02, 0.04 and 0.08 mg/L of PFOS for 7, 14, and 21 days	Immune-regulatory function in the liver was disturbed by affecting liver structure, enzyme activities (↓ACP, AKP, lysozyme, ↑MPO) via ↑ NF-κB signalling,↑ ROS	[211]
PFOA	**In vivo** zebrafish	↑ TLR2/Myd88/p65 pathway → (↓ IFN and BAFF mRNA expression → ↓ of Ig secretion) lipid metabolism disorder enhances the immune toxicity level in the spleen	[212]
PFOS	**In vitro**: mural BMDMs; human cells: THP-1 cells (10-200 nM PFOS according to the authors corresponding to PFOS-serum levels in most human subjects) [In vivo: wild type (WT) C57BL/6 J mice were injected i.p., acute: 5, 15, or 25 mg/kg/d for 5 days & chronic: 0.066 mg/kg/d for 30 days; OVA induced asthmatic exacerbation model]	THP-1 cells: ↑ NF-κB signalling; Ca^2+^–PKC-dependent pathway; ↑mRNA levels and release of TNF-α and IL-6 BMDMs: ↑ phosphorylation of NF-κB p65 and degradation of IκBα [In vivo: PFOS induced inflammation, ↑ IL-6, TNF-α, IL-1ß) tissue damage (lungs, liver, kidneys) via activation of the AIM2 inflammasome; asthmatic exacerbation, ↑IL-4, IL-1ß]	[158]
PFOA	**In vivo**: zebrafish were exposed to 0.05, 0.1, 0.5, and 1 mg/L for 21 days	↑TLR2 /Myd88/NF-κB (p65) pathway ↑ proinflammatory cytokines (IFN and IL-1β) → regulation of antibody expression and regulation of expression of other cytokines (↓IL-4) → immune disorders	[213]
**Modulation of NF-kB in different cell types (less immune relevant)**			
PFOS	In vivo: mice fed a regular (RD) or high-fat diet (HFD) and then exposed to PFOS (0, 5, and 20 mg/kg/day) for 14 days.	More serious atrophy of immune organs seen in HFD group; HFD might ↑ apoptosis caused by PFOS; no effect on NF-κB signalling pathway	[207]
PFNA	In vivo: Rats treated with PFNA or PFNA & gadolinium chloride, an inhibitor of KCs, for 14 days. In vitro: primary rat hepatocytes	↑ NF-κB, ↑ TNFα and IL-1β were involved in ↓of PPARα promoter activity.	[214]
PFOA	In vitro (cancer research): breast cancer cells MDA-MB-231	↑ NF-κB translocation into the nucleus ↑mRNA and protein levels of MMP-2/−9 → invasiveness increased	[215]
PFOS	In vitro (neurotoxicity, in this case: immunotoxicity in central nervous system): murine BV2 microglial cells	↑ NF-κB, ↑ TNF-alpha and IL-6 expression. In part via c-Jun N-terminal protein kinase, ERK and NF-κB signalling; related to neurodegenerative diseases;	[216]
PFOA	In vitro (cancer research): human colorectal cancer cell DLD-1	↑ NF-κB activity by stimulating translocation into nucleus; (↑ MMP2/9 expression → invasiveness increased)	[217]
PFOS	In vitro (neurotoxicity): HAPI rat microglia (i.e. innate immune system of the CNS)	↑ NF-κB p65 and PKC were activated, ↑TNF-α secretion via Ca^2+^-dependent PKC-NF-кB signalling	[218]
PFOA	(cardiotoxicity) fertile chicken eggs, +/− l-carnitine co-treatment	↑ p65 translocation in ED19 embryo hearts and hatchling hearts, alleviated by l-carnithine (antioxidant, NO modulatory); ↑ ROS levels	[219]
PFOA	In vitro (cancer research): A2780 human ovarian cancer cell line	↑ NF-κB signalling through ERK1/2 phosphorylation (↑of MMP-2/−9 expression associated with tumor invasion)	[220]
PFOS	In vivo (hepatotoxicity): in male mice, 10 mg/kg/day i.g. alone or with narginin for 3 weeks	↑ NF-κB activity and ↑inflammatory cytokines TNF-α and IL-6 in the liver (also Bax and caspase 3), suppressed by narginin (anti-oxidative, anti-inflammatory and anti-apoptotic)	[175]
PFOS	In vitro (neurotoxicity): astrocytes, C6 glioma cells (rat)	↑ phosphorylation and degradation of IκBα, and translocation of NF-κB p65 to the nucleus; ↑pro-inflammatory cytokines (IL-1β) via AKT-dependent NF-κB signalling pathway.	[221]
PFOA	In vitro (cancer research): in human follicular thyroid carcinoma cells (FTC133)	↑ phosphorylation of NF-κB p65 and nuclear translocation. Reversed by NF-κB inhibitor; ↑ MMP-2 and tumor invasion	[222]
PFOA	In vivo rat model of PFOA-induced patellar instability	↑ of the NF-κB signalling pathway; associated with early patellofemoral articular cartilage degeneration	[223]

For discussion of results see Results, Modulation of NF-κB regulated gene transactivation section

↑ induction, ↓ reduction, *AGS* Human gastric adenocarcinoma cell line, *BAFF* B cell-activating factor, *BMDMs* Bone marrow-derived macrophages, *CNS* Central nervous system, *EDCs* Endocrine disruptors, *HAPI* Highly aggressive proliferating immortalized rat microglia cells, *HMC-1* Cells human mastoid cell line, *IFN* Interferon, *i.g* Intragastrically, *Ig* Immunoglobulin, *IL* Interleukin, *IκBα* NF-κB inhibitor alpha, *KCs* Kupffer cells, *LPS* Lipopolysaccharide, *MMP-2/−9* Matrix metalloproteinases, *Myd88* Myeloid differentiation factor 88, *NF-κB* Nuclear factor kappa B, *OVA* Ovalbumin, *p65* NF-kappa-B p65 subunit (see also RelA), *RBL-2H3* Rat basophilic leukaemia cells, *RelA* (= p65, i.e. NF-κB subunit) REL-associated protein, *ROS* Reactive oxygen species, *SD* Sprague-Dawley, *THP-1* Human monocytic leukaemia cells, *TLR 2* Toll-like receptor 2

**Table 5:** Experimental binding/agonistic effects of PFAS to PPARγ, PPARβ/δ and PPARα (see also Involvement of PPARs section, Results)

Compound and activity	Test system (organism)	Reference
**PPARγ**		
PFBS exposure – causes elevated expression of PPARγ (b)	In vitro (HepG2)	[224]
PFOA – can activate PPARγ (e)	In vivo (mussel)	[225]
PFOA – agonistic activity towards PPARγ (f)	In vitro (HEK 293)	[226]
PFCAs – C8 to C14 showed an agonistic activity towards PPARγ, in HPA cells the activity increased from C8 to C11 and then fluctuated, in 3 T3-L1 the activity increased from C8 to C13 and then slightly fluctuated (g)	In vitro (HPA-s; HEK 293; 3 T3-L1)	[147]
PFCAs – bind to PPARγ, binding affinity increased from C4 to C11 and then decreased slightly from C12 to C18 (c)	In vitro (HepG2)	[148]
PFSAs – binding to PPARγ, binding affinity is stronger compared to PFCAs with the same carbon chain-length (c)	In vitro (HepG2)	[148]
PFOA and PFOS – bind to PPARγ, PFOA shows a higher binding affinity compared to PFOS (d)	In vitro (buffer solution)	[227]
PFOS binds to PPARγ – agonistic activity (a)	In vitro (3 T3-L1; HEK 293)	[122]
6:2 Cl-PFAES binds to PPARγ - agonistic activity (a)	In vitro (3 T3-L1; HEK 293)	[122]
8:2 Cl-PFAES binds to PPARγ - agonistic activity (a)	In vitro (3 T3-L1; HEK 293)	[122]
HFPO-TA and HFPO-DA – agonistic activity towards PPARγ (f)	In vitro (HEK 293)	[226]
PFAAs - containing carbon chain-length from C6 to C12 may alter the PPARγ activity; sulfonic acid groups showed a higher affinity to bind to PPARγ compared to carboxylic acids with the same carbon chain length (h)	In silico *(*Molecular dynamic calculations)	[228]
DONA – shows the ability to activate PPARγ (h)	In silico (Molecular dynamic calculations)	[228]
**PPARβ/δ**		
PFBA, PFHxS and PFOS – binds to PPARβ/δ (d)	In vitro (buffer solution)	[227]
PFOS binds to PPARβ/δ – agonistic activity (a)	In vitro (3 T3-L1; HEK 293)	[122]
6:2 and 8:2 Cl-PFAES bind to PPARβ/δ - agonistic activity (a)	In vitro (3 T3-L1; HEK 293)	[122]
**PPARα**		
HFPO-DA – PPARα (i) activation	Maternal and foetal liver tissue (rat)	[229]
PFBS – PPARα (b) activation	In vitro (HepG2)	[224]
HFPO-DA – PPARα (j) activation	New-born pup liver tissue (rat)	[121]
PFHxA and PFNA – strong binding to PPARα (d)	In vitro (buffer solution)	[227]
PFOS – agonistic activity towards PPARα (a)	In vitro (3 T3-L1 cells)	[122],
6:2 Cl-PFESA – strong binding to PPARα (k)	In vitro (3 T3-L1 cells)	[230]
PFBA, PFHxA, PFOA, PFHxS and PFOS – PPARα (l) activation	In vitro *(HepG2)*	[231]
PFOA – PPARα (m) activation	In vitro *(MCF-10A)*	[232]

*Abbreviations*: *3 T3-L1* Mouse preadipocyte cells, *Cl-PFAES* Chlorinated polyfluorinated ether sulfonates, *DONA* 4,8-dioxa-3H-perfluorononanoic acid, *HEK 293* Human embryonal kidney cells, *HepG2* Human hepatoma cell line, *HFPO-DA* Hexafluoropropylene oxide-dimer acid, *HFPO-TA* Hexafluoropropylene oxide trimer acid, *HPA-s* Human preadipocytes-subcutaneous, *MCF-10A* Human mammary epithelial cells, *PPAR* Peroxisome proliferator-activated receptor, *PFAA* Perfluoroalkyl acids, *PFBA* Perfluorobutanoic acid, *PFBS* Perfluorobutane sulfonate, *PFCA* Perfluorocarboxylic acid, *PFHxA* Perfluorohexanoic acid, *PFHxS* Perfluorohexane sulfonate, *PFNA* Perfluorononanoic acid, *PFOA* Perfluorooctanoic acid, *PFOS* Perfluorooctane sulfonate, *PFSAs* Perfluorosulfonic acids

**Table 6:** Modulation of calcium homeostasis by PFAS in immune-relevant cells (see also Experimental studies on modulation of calcium signalling by PFAS in immune cells section, Results)

PFAS	Study / Method	Effect	Reference
PFOS	**In vitro**: murine bone marrow-derived macrophages (BMDMs) and human cells (THP-1-derived macrophages) **In vivo*****,*** wild type (WT) C57BL/6 J mice, i.p. injection (5, 15, or 25 mg/kg/d for 5 days; 0.066 mg/kg/d for 30 days)	cytosolic Ca^2+^was increased in human and mouse macrophages (starting at 150 μM, appr. ~↑ 40% relative to control in BMDMS; in Thp-1 cells ~↑20% at 50 μmM, ~↑40% at 100 and 150 μM), concurringly with the protein level of BiP, an indicator of ER stress. The authors concluded that PFOS activates the AIM2 inflammasome in a process involving mitochondrial DNA release through the Ca^2+^-PKC-NF-κB/JNK-BAX/BAK axis	[158]
PFOS, PFOA, F-53B, PFHxS	**In vitro*****:****gene expression profiles in hBMSC at 100 nM after 7 d; perturbation of Ca2+ signalling at 1 – 100* μM *by real-time imaging*	Genes related to osteoblast differentiation, ERK1/2, TGFß and Ca2+ signalling were affected. Ca2+ transients occurred at 10 μM and 100 μM for F-53B, and at 100 μM for PFOS and PFHxS, but not PFOA.	[163]
PFOS	**In vitro**: IgE stimulated RBL-2H3 cells treated +/− PFOS for i.c. Ca^2+^-measurement (also histamine and β-hexosaminidase) **In vivo**: ovalbumin-induced active systemic anaphylaxis model using ICR mice to assess for type I hypersensitivity. After sensitization, mice were treated p.o. with PFOS (50-150 mg/kg 3 times on day 9, 11 and 13, day 14 ovalbumin i.p. → measurement of allergic symptoms (serum histamine, IgE, IgG_1_, TNF-α and rectal temperature)	increase in i.c. Ca^2+^-levels (at 500 μM with challenge at 50, 100, 500 μM), likely caused by crosslinking of FcεRI on mast cells; consequences: - the release of histamine and β-hexosaminidase and degranulation through membrane fusion was increased in IgE-stimulated mast cells; - Induction of transcription factor NF-κB; - regulation of expression of pro-inflammatory cytokines and chemokines at high concentrations (100, 500 μM);	[160]
PFDA (C10), PFUnA (C11), PFNA (C7), PFHpA (C9)	**In vitro**: IgE stimulated RBL-2H3 cells treated +/− different PFAS (100 μM) for i.c. Ca2 + −measurement (also histamine and β-hexosaminidase); Cells +/− NF-κB luciferase transporter construct	In vitro: long-chain PFDA and PFUnA increased release of histamine and β-hexosaminidase by up-regulation of i.c. Ca^2+^ levels, whereas PFNA and PFHpA did not	[111]
PFOA	**In vitro**: RBL-2H3 – mast cell like cells, expressing FcεRI, stimulated with IgE (100-500 μM PFOA)	In vitro: Increase in i.c. Ca^2+^ levels (100 – 500 μM), which caused augmented mast cell degranulation (increased release of histamine and β-hexosaminidase)	[159]
PFOA	**In vitro**: mast-cell mediated allergic inflammation HMC-1 cells treated with 25–150 μM for 24 hrs, 200 μM for 10 min (for intracellular Ca^2+)^	In vitro: increase of i.c. Ca^2+^ causing - histamine release - expression of pro-inflammatory cytokines (TNF-α, IL-1β, IL-6, and IL-8) - NF-κB, p38 mitogen-activated protein kinase (but not JNK and ERK), and caspase-1 dependent.	[128]

For discussion of results please see also Experimental studies on modulation of calcium signalling by PFAS in immune cells section, Results

*BiP* Binding immunoglobulin protein, *ERK* Extracellular signal-regulated kinases, *Fc*_*Ɛ*_*- RI* High-affinity receptor for the Fc region of IgE, *HMC-1* Human mastoid cell line, *i.c.* Intracellular, *IgE* Immunoglobulin E, *JNK* c-Jun N-terminal kinases, *NF-κB* Nuclear factor kappa B, *RBL* Rat basophilic leukaemia cells, *PKC* Protein kinase C, *hBMSC* Human bone mesenchymal stem cells

**Table 7:** Experimental studies (2018 and newer) observing induction of oxidative stress (see Results, Induction of oxidative stress and potential consequences for immune health section); modulation of NK-cell activity (see Results, Modulation of NK cell activity section) and investigation of immunoenhancement (see Results, Immunoenhancement section)

PFAS	Species	Experimental design and doses (mg/kg/day)	Effect	Significant effect level (mg/kg/d) / serum or tissue concentration	Reference
**Oxidative stress**					
PFOS	Human lymphocytes (healthy donors)	75-500 μM PFOS for 12 h (2, 4, 6, 8, 10, 12 h)	↓ cell viability ↑ ROS, lipid peroxidation, GSH depletion, apoptosis ↑ damage to cell organelles (mitochondria, lysosomes)	IC50: 163.5 μM (12 h) ROS: 75 μM (4 h)	[172]
PFOS, PFOA	C57BL/6 Mice pups, Nrf2−/− and wild-type astrocytes	75-600 μM PFOS for 24 h 400-1000 μM PFOA for 24 h	nrf2−/− astrocytes: ↑ ROS, lipid peroxidation, apoptosis ↓ GSH/GSSG ratio ultrastructural alterations of mitochondria	600 μM PFOS, 800 μM PFOA	[173]
PFOS, PFOA	Adult male C57BL/6 J mice, primary hepatocytes	0, 0.01, 0.1, 0.5, 1 mM PFOS for 24 h 0, 0.01, 0.1, 0.5, 1 mM PFOA for 24 h	↑ ROS ↑ SOD ↑ GSH ↓ CAT	1 mM PFOS, 0.5 mM PFOA 0.5 mM PFOS, 0.5 mM PFOA 0.1 mM PFOS, 0.5 mM PFOA 0.01 mM PFOS, 0.01 mM PFOA	[174]
PFOS	♂ mice (strain not specified), alone or in combination with 100 mg/kg/day naringin, *n* = 4 per group	0 or 10, intragastrically for 21 days	↑ malondialdehyde (MDA), hydrogen peroxide (H_2_O_2_) ↓ glutathione (GSH) content; superoxide dismutase (SOD) activity; NRF2 protein; gene expression of *Sod*, *Cat*, *Ho-1* ↑ liver enzymes (ALT, AST); liver weight ↑ protein levels of p53 and BAX; activity of caspase-3 ↓ protein level of BCL-2 ↑ TNF-α; IL-6	10	[175]
PFOA, PFPA, PFDA	Isolated erythrocytes	10-100 μmol/L for 3 hours	↑ malondialdehyde (MDA) ↓ glutathione (GSH) content	100 μmol/L 10 (PFOA and PFDA) 100 μmol/L (PFPA)	[176]
PFNA	Pheo-chromocytoma-12 (PC12) cells	0, 20, 50, 100 μM for 24 h	↑ MDA content ↓ total antioxidant capacity; glutathione peroxidase activity	100 μM	[177]
**NK-cell activity**					
PFOA	♀ Kunning mice, 4 dose groups, *n* = 6 per group	0, 2.5, 5, 10 from pregnancy day (PD) 1 to PD 13	↓ placental weight Interstitial oedema of placenta blood sinusoids area shrunken ↓ number of NK cells ↑ levels of BAX and cleaved caspase 3 proteins (apoptosis induction)	5 and 10 2.5 – 10 5 2.5	[178]
**Immunoenhancement**					
PFOS/PFOA	C57BL/6 M mice	0, 7, 70 mg/kg TAD PFAS in normal or OVA-induced asthmatic mice	PFOS and PFOA ↑lung inflammation, serum IgE, IL-4 and IL-13 in asthmatic mice ↑ IL-4 and IL-13 in the BALF in asthmatic mice PFOS alone: ↓serum IFNγ; ↑serum IL-13 and IL-4; ↑ IL-4 in the BALF	7 and/or 70 70 7 and 70	[184]
PFOA	BALB/c M mice	0, 10, 50 and 100 pg PFOA, intratracheal administration, in OVA sensitised mice	↑airway hyperresponsiveness and serum IL-4. ↓ serum IFNγ. ↓GR in the lung.	100 pg	[185]
PFOS, PFOA, PFHxS	In vitro, mouse bone marrow derived and human mast cells (HMC-1)	30 μM for 1 hr	↑ degranulation ↑ Ca^2+^ influx ↑LTC4 and PGD2 (lipid mediators)	PFOA and PFOS PFOA and PFOS PFHxS, PFOA and PFOS	[186]
PFOS	BALB/c F mice	10 and 100 μg/kg bw/day for 1 week, intranasal administration	↓ allergic asthma responses to dust mite, proposed due to the binding and inactivation of the dust mite antigen Der p1 ↓ *P. aeruginosa* infection, with reduced lung and blood pro-inflammatory cytokines	PFOS	[187]

Results are described in Induction of oxidative stress and potential consequences for immune health, Modulation of NK cell activity and Immunoenhancement sections

↓ reduction (suppression); ↑ increase, *BALF* Bronchoalveolar lavage fluid, *Bax* Bcl-2-associated X protein, *Ca*^*2+*^ Calcium, *CAT* Catalase, *F* Female, *GSH* Glutathione, *HMC-1* Human mast cell line, *IFNγ* Interferon gamma, *Ig* Immunoglobulin, *IL* Interleukin, *LTC4* Leukotriene C4, *M* Male, *MDA* Malondialdehyde, *NK* Natural killer, *OVA, Nrf2* Nuclear factor-erythroid factor 2-related factor 2, ovalbumin, *P. aeruginosa Pseudomonas aeruginosa*, *PBS* Phosphate-buffered saline, *PD* Pregnancy day, *PFDA* Perfluorodecanoic acid, *PFHxS* Perfluorohexane sulfonic acid, *PFNA* Perfluorononanoic acid, *PFOA* Perfluorooctanoic acid, *PFOS* Perfluorooctane sulfonic acid, *PFPA* Perfluoropentanoic acid, *PGD2* Prostaglandin D2, *PND* Postnatal day, *SOD* Superoxiddismutase, *ROS* Reactive oxygen species, *TAD* Total administered dose, *TNFα* Tumor necrosis factor alpha

## Abbreviations

AIM2: Absent in Melanoma 2 (inflammasome)
AOP: Adverse Outcome Pathway
ATSDR: U.S. Agency for Toxic Substances and Disease Registry
BaP: Benzo[a]pyrene
BPA: Bisphenol A
Ca^2+^: Calcium ion
CAR: Constitutive Activated Receptor
CAT: Catalase
Cl-PFAES: Chlorinated polyfluorinated ether sulfonates
Cl-PFOS: Chlorinated polyfluorooctane sulfonate
COVID-19: Coronavirus Disease caused by the SARS-CoV-2 virus
COX-2: Cyclooxygenase-2
CRAC: Calcium Release-Activated Calcium Channel
EC: European Commission
ECHA: European Chemicals Agency
EFSA: European Food Safety Authority
EOGRTS: Extended-One Generational Reprotoxicity Toxicity Study
ER: Endoplasmatic Reticulum
F-53B: 6:2 chlorinated polyfluorinated ether sulfonate
FA: Fatty Acid
GenX chemicals: HFPO dimer acid and its ammonium salt
GSH: Glutathione
GPx: Glutathione Peroxidase
HBM4EU: Human Biomonitoring for Europe (The European Human Biomonitoring Initiative)
HFPO: Hexafluoropropylene oxide
HFPO-DA: HFPO-dimer acid
KCs: Key characteristics
IFNɣ: Interferon Gamma
IATA: Integrated Approaches to Testing and Assessment
Ig: Immunoglobulin
IL: Interleukin
Ip_3_R: Inositol 1,4,5-trisphosphate Receptor
LDL: Low Density Lipoprotein
LRTI: Lower Respiratory Tract Infections
MDA: Malondialdehyde
N: Number
NAMs: New Approach Methodologies
NFAT: Nuclear Factor of Activated T-cells
NF-κB: Nuclear Factor kappa B
NK: Natural killer (cells)
NLRP3: NOD-, LRR- and pyrin domain-containing protein 3 (inflammasome)
Nrf2: Nuclear factor erythroid 2-related factor 2
NTP: National Toxicology Program
OECD: Organisation for Economic Co-operation and Development
OVA: Ovalbumin
PAHs: Polycyclic Aromatic Hydrocarbons
PFAS: Per- and Polyfluoroalkyl substances
PFBS: Perfluorobutane sulfonate
PFCAs: Perfluoroalkyl Carboxylic Acids
PFDA: Perfluorodecanoic acid
PFDoDA: Perfluorododecanoic acid
PFHxA: Perfluorohexanoic acid
PFHxS: Perfluorohexane sulfonic acid
PFMOAA: Perfluoro-2-methoxyaacetic acid
PFMOBA: Perfluoro(4-methoxybutanoic) acid
PFMOPrA: Perfluoro-3-methoxypropanoic acid
PFOA: Perfluorooctanoic acid
PFOS: Perfluorooctanesulfonic acid
PFPA: Perfluoropentanoic acid
PFSAs: Perfluoroalkane Sulfonic Acids
PFUnDA: Perfluoroundecanoic acid
PKC: Protein Kinase C
POPs: Persistent Organic Pollutants
PPARs: Peroxisome Proliferator-Activated Receptors
RCT: Randomised Controlled Trials
ROS: Reactive Oxygen Species
RPFs: Relative Potency Factors
RXR: Retinoid X Receptor
RYR1: Ryanodine Receptor
SERCA: Sarcoplasmic Reticulum Ca^2+^-ATPase
SOCE: Store-operated Ca^2+^ entry
SOD: Superoxide Dismutase
T1D: Type 1 Diabetes
Tc cells: cytotoxic T-cells
TDAR: T-cell Dependent Antibody Response
TDI: Tolerable Daily Intake
TG: Test Guideline
TGF-ß: Transforming Growth Factor beta
Th cells: T-helper cells
TIAR: T-cell Independent Antibody Response
TNF-α: Tumor Necrosis Factor alpha
TWI: Tolerable Weekly Intake
U.S. EPA: United States Environmental Protection Agency
WBC: White Blood Cell
WHO: World Health Organization
